# A detection method for synchronous recognition of string tomatoes and picking points based on keypoint detection

**DOI:** 10.3389/fpls.2025.1614881

**Published:** 2025-07-24

**Authors:** Linqiang Deng, Rongting Ma, BaoFan Chen, Guozhu Song

**Affiliations:** College of Software, Shanxi Agricultural University, Taigu, China

**Keywords:** keypoint detection, YOLOv8, string tomato, picking point, PSA mechanism

## Abstract

In the greenhouse environment, factors such as variable lighting conditions, the similarity in color between fruit stems and background, and the complex growth posture of string tomatoes lead to low detection accuracy for picking points. This paper proposes a detection method for the synchronous recognition of tomatoes and their picking points based on keypoint detection. Using YOLOv8n-pose as the baseline model, we constructed the YOLOv8-TP model. To reduce the computational load of the model, we replaced the C2f module in the backbone network with the C2f-OREPA module. To enhance the model’s accuracy and performance, we introduced a PSA mechanism after the backbone network. Additionally, to strengthen the model’s feature extraction capabilities, we incorporated CGAFusion at the end of the Neck, which adaptively emphasizes important features while suppressing less important ones, thereby enhancing feature expressiveness. Experimental results show that the YOLOv8-TP model achieved an accuracy of 89.8% in synchronously recognizing tomatoes and picking points, with an inference speed of 154.7 FPS. The YOLOv8n-pose model achieves an inference speed of 148.6 FPS. Compared to the baseline model, YOLOv8-TP improved precision, mAP@.5, mAP@.5:.95, and F1-score by 0.6%, 1%, 2%, and 1%, respectively, while reducing model complexity by 8.1%. The Euclidean distance error for detecting picking points was less than 25 pixels, and the depth error was less than 3 millimeters. This method demonstrates excellent detection performance and provides a reference model for detecting string tomatoes and their picking points.

## 1 Introduction

China is one of the countries with the largest area for string tomato cultivation and the highest production. According to statistics, the production of string tomatoes in China increased from 6.2 million tons in 2022 to 8 million tons in 2023, and it is expected that by the end of 2024, the production will exceed 11 million tons (China Report Hall Network, 2024). However, string tomato harvesting faces several challenges, including ensuring harvest integrity, low manual efficiency, high labor intensity, and a shortage of skilled labor. With the development of artificial intelligence, automated harvesting technology has rapidly emerged (Fujinaga et al., 2021; Matsuo et al., 2021). In string tomato harvesting, detecting both the tomatoes and their picking points is crucial and relies on computer vision technology and deep learning models (Zhang et al., 2020; Wang and Zhu, 2023).

In natural environments, string tomato clusters are often obscured by leaves, branches, and other tomato clusters (Berenstein et al., 2010). Additionally, the color of the fruit stems is similar to that of leaves, and their complex posture poses challenges in locating picking points. Therefore, the core of developing string tomato harvesting robots lies in creating precise and efficient algorithms for the simultaneous detection of string tomatoes and picking points. At present, there are two latest methods for locating picking points. One is based on instance segmentation, such as (Rong et al., 2025), who proposed an enhanced dual-stream architecture algorithm that combines RGB and depth features for tomato organ instance segmentation. In their research, they attempted to combine RGB and depth information to achieve more accurate tomato organ instance segmentation. By using a dual-stream architecture, it can better utilize complementary information from different modalities. However, the limitation of this algorithm is the high computational cost. Simultaneously processing RGB and depth images requires a significant amount of computational resources, which may limit the real-time performance of the algorithm in practical applications. For example, in large-scale tomato plantations, if the algorithm’s image processing speed is not fast enough, it will affect the picking efficiency. Another limitation is the sensitivity to occlusion. When tomatoes or their fruit stalks are severely obstructed by leaves or other objects, for instance, segmentation results may be inaccurate, leading to incorrect recognition of cutting points. Another method is (Zhang et al., 2022)’s tomato skewer 3D pose detection method based on a key-point detection network, which focuses on detecting the keypoints of tomato skewers to estimate their 3D pose. By training a keypoint detection network, the goal is to accurately locate the key positions of tomatoes and their fruit stalks. However, this method may lead to misjudgment of keypoints due to complex backgrounds, and may also encounter challenges when encountering occlusions.

In addition, research on identifying and locating tomato clusters and picking points can be roughly divided into traditional image processing methods (TIPM) and deep learning methods (DLM). TIPMs detect fruits based on pre-defined handcrafted features such as fruit shape, color, and geometric characteristics. They then predict locations based on geometric features (such as fruit contours and centroids) or identify stems based on their positional relationship with fruits to locate picking points. In related studies, (Zhou et al., 2023). improved the YOLACT++ model to identify and segment key structures such as result branches, fruit stems, and fruit clusters; they designed a method for selecting low-collision regions of interest (ROI) based on structural constraints and range re-selection, using the centroid of this region as the picking point. (Xu et al., 2023). proposed a feature-enhanced recognition deep learning model named YOLO v4-SE that combines multi-channel inputs from RGB and depth images to identify grapes while simultaneously inferring picking points above the predicted grape bounding boxes. (Li et al., 2024). developed a system with object detection and instance segmentation capabilities along with a picking point localization algorithm; this system derives a skeleton line for stem regions based on segmented images and develops an algorithm to determine optimal picking point coordinates. (Zhang et al., 2023). utilized image processing techniques within the YOLOv5-GAP framework to identify the top vertices of grape regions based on grape detection. (Bai et al., 2023). proposed a method that combines Hough circle detection, spatial symmetric spline interpolation, and geometric analysis for estimating clustered tomato flower stems, contour fitting, and selecting point localization.

DLMs can learn higher-level and more complex semantic features, thereby improving detection accuracy, robustness, and generalization ability. They have been widely applied in scenarios such as pest recognition, weed identification, and monitoring crop growth conditions. In research focused on detecting fruits and stems, (Zhang et al., 2024). addressed challenges such as leaf occlusion and small target sizes that hinder accurate determination of string tomato picking points by proposing a YOLOv8n-DDA-SAM model that incorporates a semantic segmentation branch into object detection to achieve accurate detection and calculation of picking points with an accuracy rate of 85.90%. (Yan et al., 2023). introduced a Si-YOLO-based deep learning algorithm for recognizing and locating string tomato picking points in unstructured environments. This method enhances dataset accuracy by combining object detection algorithms with attention mechanisms while utilizing GANs alongside traditional image data augmentation techniques to more accurately locate string tomato picking points and improve model generalization. (Zhu et al., 2023). used grape clusters and stems as two target categories employing the YOLOv5m-CFD model for object detection; the midpoint of the stem prediction box was marked as the picking point. (Rong et al., 2023). proposed an improved Swin Transformer V2 semantic segmentation model along with a picking point recognition algorithm based on connections between tomato fruits, sepals, and stems to address challenges in identifying picking points for mature tomatoes in complex environments. The sea ice recognition study by (Zhou et al., 2023). uses YOLACT to address the core challenges of dense target adhesion and light interference for instance segmentation in complex environments. (Wang et al., 2025). solved the problem of insensitivity of traditional methods to target location, structural differences and edge details in high-resolution images by means of Dual-path Transformer (DPT) and Unit Fusion Module (UFM).

The aforementioned methods exhibit limitations in synchronizing the detection of string tomatoes and their picking points; moreover, TIPMs require significant time for manual feature selection and design while having limited adaptability to complex scenes—struggling with diverse images under specific scales or angles. Additionally, these models still possess considerable computational demands which pose challenges for deploying models on mobile terminals, affecting detection speed. In practical robotic harvesting processes, factors such as uneven lighting conditions and varying growth postures of string tomato clusters impact harvesting efficiency; thus, the vision system of string tomato harvesting robots must achieve precise localization of both tomatoes and picking points to minimize losses during harvesting while reducing damage to the fruit clusters (Klaoudatos et al., 2019). This study proposes an improved keypoint detection method based on YOLOv8n-Pose capable of simultaneously detecting string tomatoes and their picking points. This end-to-end model not only addresses the issues of insufficient robustness and low detection rate of traditional methods. Through a series of improvements, such as designing specific modules and introducing relevant mechanisms, which aim to overcome the limitations of traditional methods in handling occlusion and environmental variability, it also effectively improves the localization accuracy of string tomatoes and their picking points, providing a more accurate and efficient solution for string tomato harvesting. In the agricultural computer vision domain, studies including 3D point cloud-based phenotyping for Chinese Cymbidium seedlings and four-stream radiative transfer models for row crops primarily address macro-scale feature extraction or physical modeling. In contrast, this work emphasizes micro-scale robotic interaction by integrating keypoint detection into YOLOv8 for simultaneous tomato localization and picking-point identification. Unlike methods relying on multi-sensor data, our lightweight architecture achieves real-time performance through end-to-end learning, rendering it suitable for dynamic greenhouse environments with occlusions and variable lighting conditions. While vegetation cover estimation models typically adopt pixel-level dichotomy, our approach utilizes adaptive feature fusion (CGAFusion) to address color similarity challenges between fruit stems and backgrounds, thereby enhancing localization precision in complex scenes. The details are as follows: The model realizes the simultaneous detection of string tomatoes and picking points, which reduces the separation of detection and localization tasks in the traditional method, thus improving the detection speed and accuracy. This synchronized detection not only reduces false alarms but also improves the model’s adaptability in complex scenes. Since string tomatoes are often occluded by leaves, branches, and other tomato fruits, it is difficult for existing conventional image processing methods to effectively detect occluded fruits and picking points. In this study, these occlusion problems are overcome by an enhanced feature extraction mechanism, which improves the accurate recognition of string tomatoes in complex environments. Considering the diverse growth postures and uneven light conditions of string tomatoes, the proposed model possesses strong environmental adaptability. It can maintain high detection accuracy under different light conditions and complex morphology of tomato clusters, thus ensuring the minimization of losses during picking.

## 2 Materials and methods

### 2.1 Data sample collection

The data samples were collected from the Tomato Town located in Taigu District, Jinzhong City, Shanxi Province. Due to the close proximity and high density of the shooting scenes, specific requirements were placed on the selection of camera resolution and focal length. The data samples were captured using a mobile phone’s rear camera, which has a resolution of 2778×1284 pixels and a 12-megapixel sensor. The telephoto camera has a focal length of 77 millimeters, enabling the acquisition of high-quality, distortion-free image data from multiple positions and angles.

**Figure 1:**
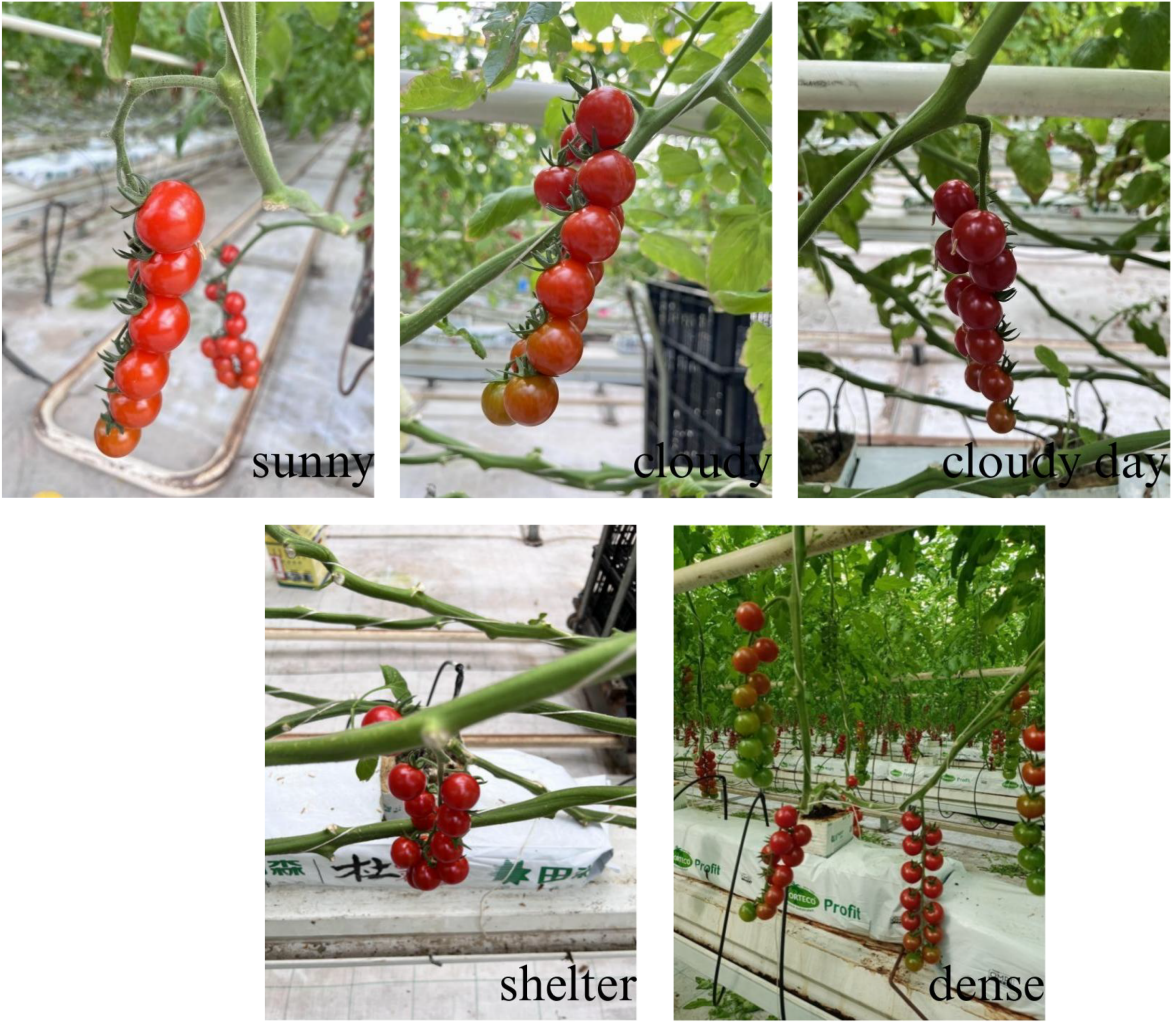
Photographs of string tomatoes in their respective conditions.

In this study, we constructed the Tomato-P dataset to train a model capable of simultaneously detecting string tomatoes and their picking points. The data was collected between July 15 and July 31, 2022, during which most string tomatoes in the greenhouse were in the ripening stage, suitable for harvesting and yield estimation. Preliminary research indicated that images captured under different lighting intensities exhibited variations; therefore, we chose to conduct the data collection from 7 AM to 7 PM, encompassing various weather conditions such as sunny and overcast days, as well as different lighting scenarios like front lighting and backlighting (Qin et al., 2021). This approach allowed for multi-angle captures of string tomatoes to obtain images under natural lighting conditions. The principle is shown in **Figure 1**.

**Figure 2:**
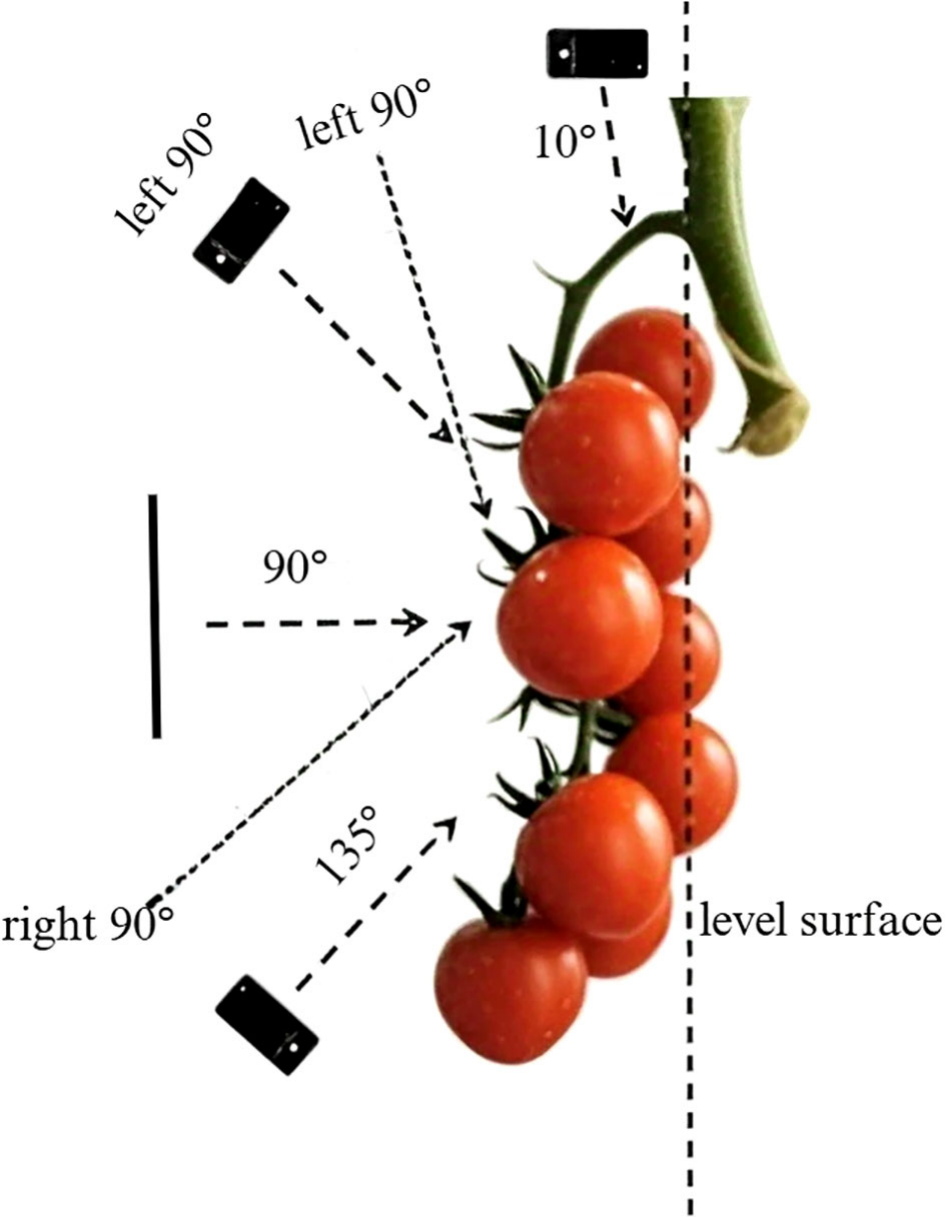
Schematic diagram of string tomato shooting angle.

To enhance data diversity, we established a baseline using the plane of string tomato fruits and tomato ridges, capturing images at angles of 10°, 45°, 90°, and 135°. The principle is shown in **Figure 2**. Additionally, we took one photograph from both the left and right sides of each string tomato cluster, ensuring that each cluster was photographed six times. The distance between the camera and the plants was maintained between 10 cm and 30 cm to ensure optimal imaging conditions. Based on the spacing between plants, the camera was positioned every 20 cm along the rows of string tomatoes to ensure that each image field contained a new cluster. The collected images were uniformly stored in JPG format with dimensions of 3024×4032 pixels. This method of having the fruit occupy a large portion of the image is helpful in multiple ways for fruit detection and harvesting. It enhances feature extraction, enabling the model to better capture details like fruit texture and color, thus improving the robustness of detection under various lighting conditions and occlusions. It also aids in model training by providing a high-quality representation of the target, helping the model distinguish fruits from surrounding foliage or branches, and can be used for pre-training or fine-tuning to boost baseline performance. For harvesting, it allows the model to focus on picking points and learn accurate localization through close-up images. Additionally, it can simulate realistic scenarios via data augmentation for better model generalization, helps the model address occlusion challenges by emphasizing features of partially visible fruits, and is suitable for specific use cases in certain greenhouse or controlled environments.

### 2.2 Data sample collection

After conducting field investigations and sampling at the “Tomato Town” in Shanxi, we organized and annotated the images of string tomatoes to construct a dataset for string tomato detection. We used the open-source annotation software LabelMe to perform bounding box annotations on the string tomato image data, with the annotation format being JavaScript Object Notation (JSON). The annotation process followed these standards:

1. Bounding Box Annotation: The areas of string tomato clusters were marked using “rectangle boxes” that tangentially fit the boundaries of the clusters. Labels were created based on ripeness and whether the picking points were obscured, categorizing them into four types: ripe (R), ripe but with obscured picking points (R-S), transitioning from unripe to ripe (GR), and unripe (G). Notably, since unripe tomatoes (GR and G categories) are not typically harvested, this study did not differentiate occluded vs. non-occluded picking points for these classes. The detailed distribution of images per category under different lighting conditions is shown in **Table 1**.

**Table 1:** Distribution of tomato cluster images by ripeness category and lighting condition.

Tomato category	Sunny(images)	Cloudy(images)	Cloudy day (images)	Shelter(images)	Dense(images)	Total(images)
R	609	435	348	209	139	1740
R-S	334	239	191	115	76	955
GR	236	169	135	81	54	675
G	103	74	59	35	24	295

**Figure 3:**
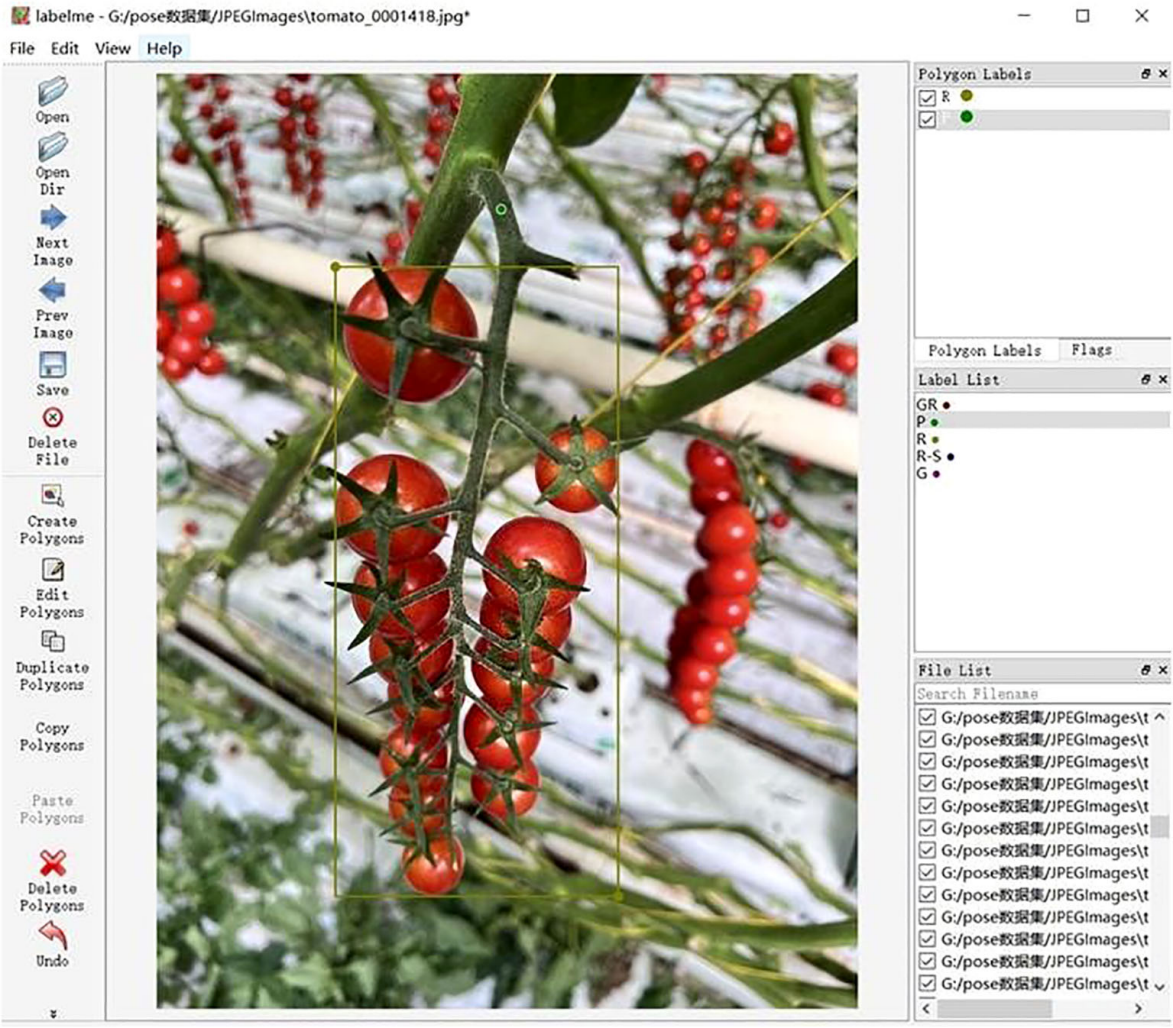
Model diagram for labeling.

2. Point Annotation: Picking points were marked using “points,” which needed to be accurately located at the center of the fruit stem. When a picking point was completely obscured by leaves or branches, its actual position could not be accurately annotated, and the approximate location was estimated based on empirical knowledge. This may introduce annotation biases and affect the model training outcomes. An example of the annotation process is illustrated in **Figure 3**.

The annotation format is shown in Equation (1):


(1) $$\begin{array}{c} \text{LabelTomato}-\text{P }\\ =(\text{class};\text{ xbox};\text{ ybox};\text{ w};\text{ h};\text{ xP};\text{ yP};\text{ visible}-\text{P}) \end{array}$$

In this study, the annotation format for the string tomato detection dataset is defined as follows:

The class represents the object category. (xbox, ybox) denotes the coordinates of the center point of the bounding box around the string tomato cluster. (w, h) indicates the width and height of the bounding box. (xP, yP) represents the coordinates of the picking point. When the value of visible-P is 2, it indicates that the keypoint is visible; when the value is 1, it indicates that the keypoint is occluded and not visible.

This structured annotation format ensures precise localization and classification of both string tomatoes and their respective picking points, facilitating effective training and evaluation of detection models.

This study includes a dataset with 3,665 annotated instances, which is divided into training, validation, and test sets in a ratio of 8:1:1. Specifically, the dataset comprises 2,932 images for the training set, 367 images for the validation set, and 366 images for the test set.

### 2.3 Experimental environment and equipment

The experimental equipment configuration parameters used in this study are shown in **Table 2**. The processor is the 13th generation Intel (R) Core (TM) i7 - 13700K with 24 threads. The graphics card is NVIDIA GeForce RTX 3090, and the graphics driver version is NVIDIA SMI 535.161.07. The memory is DDR5 64GB, and the operating system is Ubuntu 22.04.3 LTS. The depth camera is Intel RealSense D435i, with a depth image resolution of 1280x720 and a maximum frame rate of 90 frames per second. The development language used is Python 3.9.7, and the configured environment includes CUDA version CUDA_11.5.r.5 and Anaconda version conda 4.10.3. In the model of this study, the optimizer adopted is SGD (Stochastic Gradient Descent). It plays a crucial role in the training process by adjusting the model parameters based on the gradients calculated from the training data. In our experiment, the learning rate is set to 0.01, and the optimizer uses this learning rate to update the model weights in each iteration to minimize the loss function. Regarding the loss function, the model combines multiple loss components. There is a classification loss, which is a cross-entropy loss used to distinguish different classes of objects (such as tomatoes and the background in this study). There is also a regression loss for predicting the bounding boxes of objects, which is a variant of the L1 or L2 loss like the Smooth L1 loss. Additionally, for the keypoint detection task, there is a keypoint loss designed to minimize the error between the predicted keypoints and the ground truth keypoints. In our experiment, these loss components work together to guide the training of the model and evaluate its performance. Concerning the training strategy, an early stopping mechanism is implemented. Specifically, if the validation loss does not decrease for 5 consecutive epochs, the training process will be stopped. This helps to prevent overfitting and saves computational resources. By monitoring the validation loss during training, we can ensure that the model has good generalization ability and does not overfit the training data. The experimental parameter batch is 32, and the parameter learning rate is 0.01. To prevent data overfitting, the epoch is set to 200, and the rest are default values.

**Table 2:** Configuration parameters of the experimental environment.

Configuration names	Parameters
Processor	13th Gen Intel(R) Core(TM) I7-13700K×24
Display Card	NVIDIA GeForce RTX 3090
Graphics Card Driver	NVIDIA-SMI 535.161.07
RAM	64G
Development language	Python 3.9.7
Deep Learning Framework	Pytorch
Depth camera	Intel RealSense D435i
CUDA	cuda_11.5
Anaconda	conda 4.10.3
GPU memory	RTX 3090(24 G)
Training duration	1.50 hours

### 2.4 Constructing the YOLOv8-TP model

YOLOv8n-Pose extends the YOLOv8n architecture for real-time object detection and keypoint estimation. Its backbone employs a CSP (Cross-Stage Partial Network) design, an optimized deep-learning architecture that enhances model efficiency and accuracy while reducing computational and memory overhead. The backbone integrates C2f (Concatenate, Two Fusion) modules, which aggregate multi-scale features via bottleneck structures with skip connections, ensuring computational efficiency while preserving spatial information. The neck network utilizes either FPN(Feature Pyramid Network) or PAN(Path Aggregation Network) for multi-scale feature fusion, enabling effective detection of objects of varying sizes. The head module includes a pose estimation branch for predicting keypoints, such as stems and cutting points, alongside bounding box and class prediction branches. However, YOLOv8n-Pose exhibits limitations in occlusion robustness and lacks global context awareness for detecting complex object relationships. To address these, we propose the YOLOv8-TP model, which incorporates the C2f-OREPA module, integrates the PSA(Partial Self-Attention) mechanism, and inserts CGAFusion to enhance the baseline YOLOv8n-Pose architecture.

### 2.5 Design of the C2f-OREPA module

**Figure 4:**
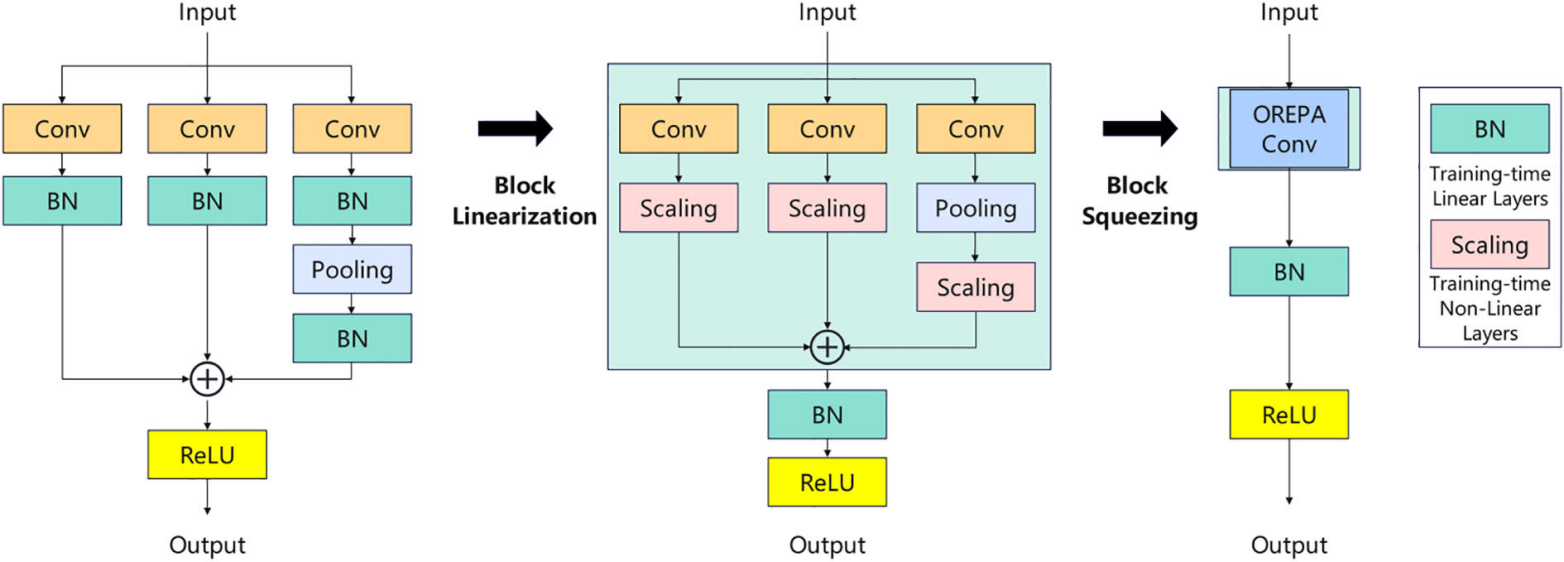
Principle of C2f-OREPA.

Aiming at the problem of low training efficiency and limited feature diversity caused by the BN(Batch Normalization) layer in the traditional C2f module, it is replaced with the C2f-OREPA module (Hu et al., 2022; Rongli et al., 2024), which achieves efficient and high-performance detection through the following three-phase improvement. Linearization modification, removing the nonlinear BN in the bottleneck layer and replacing it with a learnable linear scaling layer (Scaling), which retains the feature distribution flexibility and avoids the suppression of dense targets (e.g., string tomato) by over-normalization. Training stability is enhanced by adding independent BN layers at the end of branches to prevent gradient anomalies and ensure convergence. OREPA reparameterization, which compresses the multi-branch structure into a single convolutional layer, retains the multipath optimization during training and merges it into a single branch during inference to reduce latency. Finally, it is introduced into the C2f module, named the C2f-OREPA module. By reparameterizing the complex structure into a single convolutional layer, the training cost is greatly reduced while maintaining high performance, which helps to improve the accuracy of target detection. The principle is shown in **Figure 4**.^1^

### 2.6 Adding the partial self-attention mechanism

In the optimization process of the C2F module, the attention mechanism has become a core element for breaking through performance bottlenecks. Traditional standard attention mechanisms, such as Squeeze-and-Excitation (SE) and Convolutional Block Attention Module (CBAM), have enhanced the model’s response to key features to a certain extent by dynamically adjusting channel-wise or spatial dimension weights. However, when dealing with complex scenes, these mechanisms exhibit significant limitations: the SE mechanism solely focuses on channel relationships and lacks effective modeling of spatial information. When facing tomatoes with intertwined branches and leaves, it struggles to accurately capture spatial position and shape information, thus easily missing the characteristics of occluded fruits and leading to false negatives; although CBAM integrates channel and spatial attention, its computational overhead increases nonlinearly with input size. For instance, when processing tomato images with varying growth stages and significant size differences, the processing speed is insufficient to meet the real-time detection requirements of agricultural production. In contrast, the Efficient Partial Self-Awareness (PSA) mechanism (Wang et al., 2024) improves model performance while reducing computational complexity and memory usage. By introducing PSA into target detection models like YOLOv8 and optimizing the model’s main architecture with it, PSA helps the model better extract image features in the backbone, especially for targets of varying scales and shapes, and captures their feature information more accurately.

**Figure 5:**
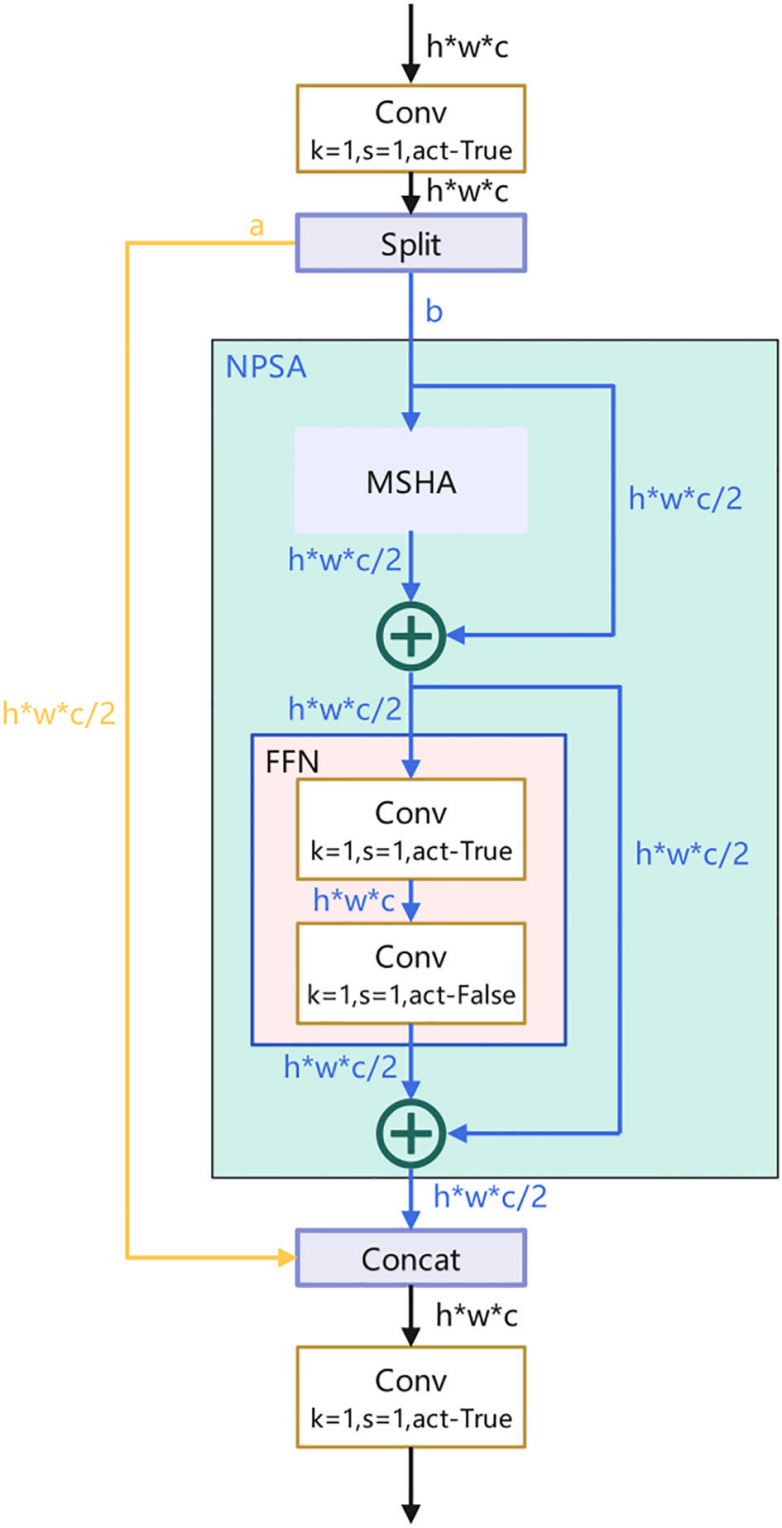
Structure of PSA attention mechanism.

Specifically, after 1×1 convolution, the feature map is evenly divided into two parts. One part is fed into an NPSA block consisting of a multi-head self-attention module (MHSA) and a feed-forward network (FFN), where the MHSA captures the dependencies between different positions to provide global information for the model, and the FFN further processes and transforms this information to better adapt to the needs of the target detection task. The other part of the features is directly processed by 1×1 convolution and then connected and fused with the part processed by the NPSA block. This design takes advantage of the global modeling capability of the self-attention mechanism and avoids the huge computational overhead associated with self-attention computation on the entire feature map. The structure of the PSA attention mechanism is shown in **Figure 5**.^2^

### 2.7 Insertion of content guided attention

**Figure 6:**
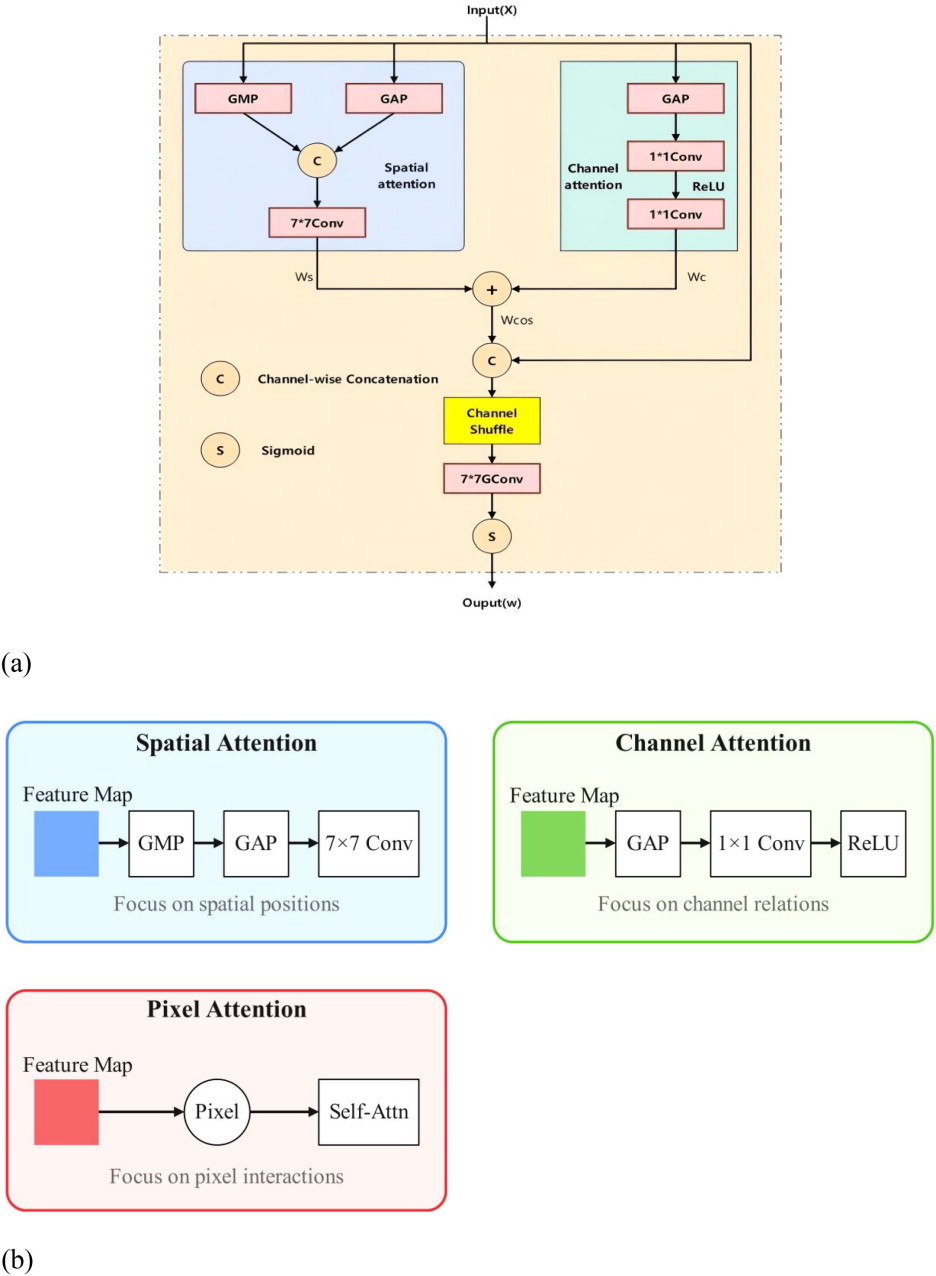
**(a)** Schematic diagram of content-guided attention. **(b)** Spatial, Channel, and Pixel Attention Supplement Maps.

By inserting a Content Guided Attention (CGA) mechanism in the Neck section, channel-specific Spatial Importance Maps (SIMs) can be generated and channel attention and spatial attention can be fused to emphasize more useful information encoded in the features for information interaction and efficient gradient flow. CGAFusion (Content Guided Attention) (Chen et al., 2023) implements a feature fusion network, which combines spatial attention, channel attention, and pixel attention mechanisms for adapting and fusing two input feature maps x and y. In this way, the network prioritizes critical features and suppresses irrelevant ones, enhancing feature representation. Among them, (1) Spatial Attention mechanism (Spatial Attention), this is usually used to enhance the model’s attention to specific regions in the image. By calculating the results of average pooling and maximum pooling and combining them for input to the convolutional layer, an attention weight map can be obtained, which assigns a weight to each spatial location of the input feature map, emphasizes important features and suppresses unimportant features; (2) Channel Attention, which aggregates the spatial information of each channel through global average pooling, and then enhances the expression of features through global average pooling. Spatial information for each channel and then learns the importance weights for each channel through a network containing two convolutional layers. This mechanism is commonly used for feature recalibration, allowing the network to adaptively emphasize informative features and suppress irrelevant ones. In this way, channel attention helps to improve the model’s ability to represent the input data, thus improving performance in a variety of visual tasks; (3) Pixel-level attention mechanism (Pixel Attention), which processes the results of the stitching of the input feature maps and the first attentional feature maps through the convolutional layers. This mechanism aims to assign a weight to each pixel, emphasizing the important pixels and suppressing the unimportant ones. In this way, pixel attention helps to enhance the model’s ability to capture key information in the image and improve the performance of processing image-related tasks. Ultimately, fusion results are generated by considering these attention mechanisms, where pixel attention weights are used to adjust the combination of input feature maps, allowing the model to focus more on features that are more discriminative to the task. This fusion strategy can improve the performance of the model in computer vision tasks such as image classification, target detection, and semantic segmentation. A schematic of content-guided attention (CGA) is shown in **Figure 6a** ^3^. In addition, we provide a simple illustration of the spatial, channel, and pixel attention mechanisms in **Figure 6b**.

### 2.8 YOLOv8-TP model

**Figure 7:**
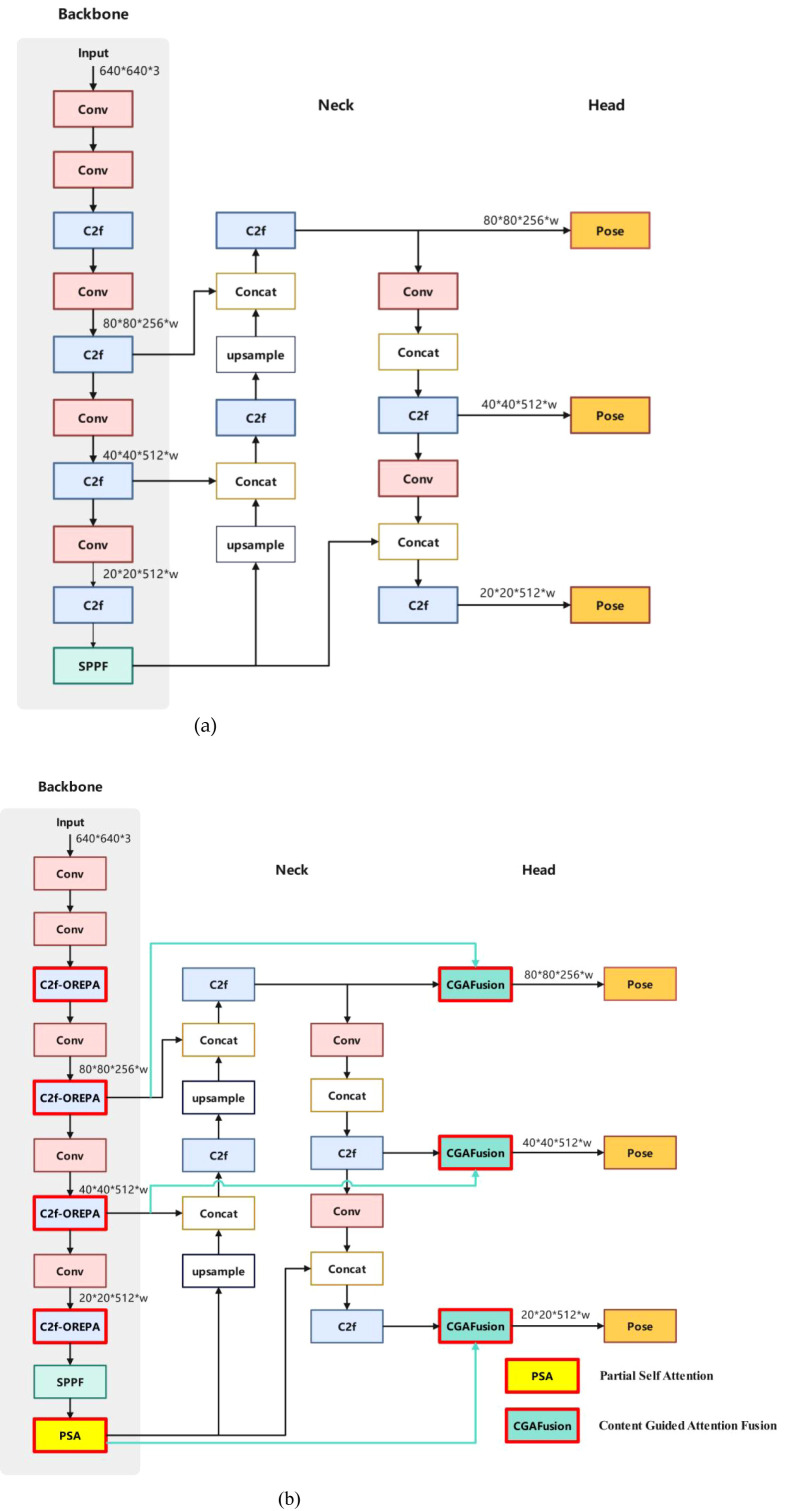
Structure of YOLOv8n-Pose and YOLOv8-TP RP. **(a)** Structure of YOLOv8n-Pose. **(b)** Structure of YOLOv8-TP.

In this paper, the YOLOv8-TP model is constructed by making innovative improvements to the base model YOLOv8n-pose(its structure is shown in **Figure 7b**), and the model structure is shown in **Figure 7a**)

The specific execution process of the YOLOv8-TP model is as follows:

1 Before inputting the image into YOLOv8-TP, any size of the image is resized to 640 × 640 × 3. The input image undergoes feature extraction by the backbone network, and a series of feature maps with different scales are obtained;
2. Further feature learning and compression of the feature maps is performed using the OREPA structure;
3. Add the PSA attention mechanism after the backbone network;
4. Input the feature maps processed by the PSA attention mechanism into CGAFusion to calculate the weights, and then combine them using the weighted sum method.
5. The final obtained features are fed into the detection head (detection head) for target classification and bounding box regression;
6. For each scale of the feature map, the bounding box, as well as the keypoints, are screened by the non-maximum suppression (NMS) algorithm to remove the redundant detection results;
7. Finally, the bounding box processed by NMS is restored to the original image size, and the final target detection results are output.

### 2.9 Assessment of indicators


(2) $$\text{P}=\frac{\text{TP}}{\text{TP}+\text{FP}}$$

(3) $$\text{R}=\frac{\text{TP}}{\text{TP}+\text{FN}}$$

(4) $${\text{F}}_{1}=\frac{2\times \text{P}\times \text{R}}{\text{P}+\text{R}}$$

The evaluation metrics of the detection model use parameters (Params) and giga floating point operations per second (Flops) to evaluate the complexity of the model. The detection speed of the model is measured in frames per second (FPS). The average precision is represented using mAP and its value is calculated from the Precision and Recall of the prediction model; the average accuracy in this study can be represented by the AP value, which is the area enclosed by the Precision-Recall curve and is calculated using Precision (P), Recall (R), and Fl score using (Equations 2-4) as shown below:

Let the target to be detected be a positive class and the others be negative classes, then: TP is the positive class predicted to be positive; FP is the negative class predicted to be positive; FN is the positive class predicted to be negative. Finally, the samples were fed into the model and executed 500 times to derive the detection speed (FPS) of the model.

In addition, considering the detection of tomato bunch picking points, the detected picking points will have a certain error with the actual picking points. So in the picking point detection experiment, we choose the pixel Euclidean distance between the predicted picking point and the real picking point as the index to evaluate the accuracy of the picking point (Hu et al., 2022). The formula for calculating the pixel Euclidean distance between the detected picking points and the actual picking points is presented in (Equations 5-7). In equations, x and y signify the normalized coordinates of the actual picking points in the horizontal and vertical directions, respectively. Correspondingly, x’ and y’ denote the normalized coordinates of the detected picking point in the horizontal and vertical directions. The variable w represents the image resolution in the horizontal dimension, and h represents that in the vertical dimension. D_X_ and D_Y_ are the pixel distances between the detected picking points and the actual picking points in the horizontal and vertical directions, respectively, while O represents the pixel Euclidean distance between the detected picking points and the actual picking points.


(5) $${\text{D}}_{\text{X}}=\text{w}(\text{x}-\text{x}")$$

(6) $${\text{D}}_{\text{Y}}=\text{ h}(\text{y}-\text{y}")$$

(7) $$\text{O}=\sqrt{{\text{D}}_{X}^{2}+{\text{D}}_{Y}^{2}}$$

## 3 Results and analysis

### 3.1 Comparison of model detection effect before and after improvement

In order to verify the advantages of the YOLOv8-TP model proposed in this study on the string tomato as a whole as well as the recognition effect of keypoints, comparative experiments are conducted on the recognition effects of the YOLOv8n-pose model and the YOLOv8-TP model. In the comparison test, the YOLOv8n-pose model and YOLOv8-TP model are trained based on the same training set and test platform, and the recognition effect is evaluated based on the same test set after training.

**Figure 8:**
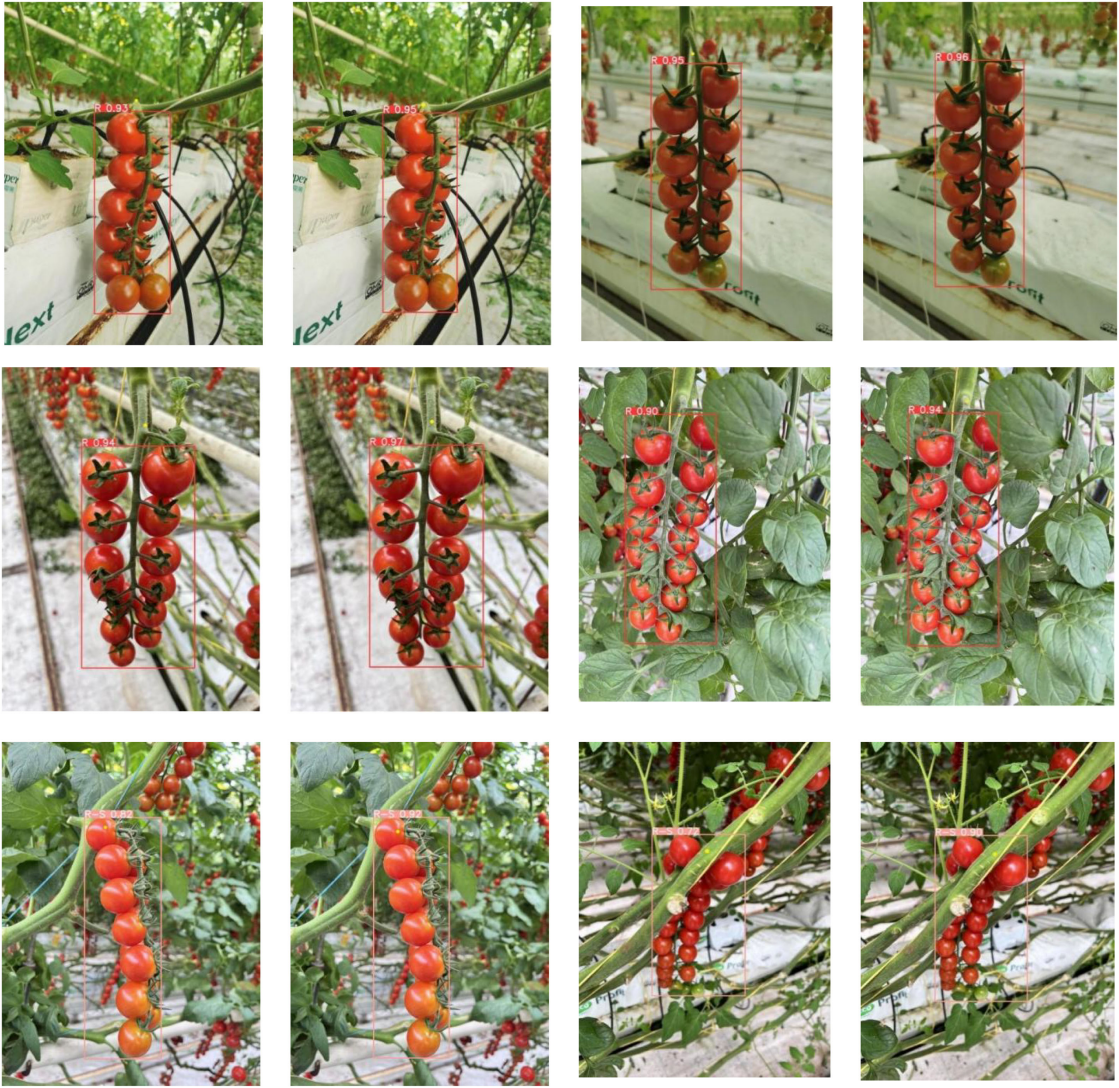
Comparison of model detection effect before and after improvement. (YOLOv8n-Pose on the left and YOLOv8-TP on the right of each group in the figure).

As shown in **Figure 8**, in the overall recognition process of samples with multiple angles, different lighting conditions, and complex growth and hidden fruit stems, the two models exhibit significant differences and fluctuations. When the tomato skewers are clearly visible, the confidence level of the YOLOv8-TP model is slightly better than that of the YOLOv8n-pose model; When tomato skewers are in a shaded environment, the confidence level of the former is significantly higher than that of the latter. In addition, the YOLOv8-TP model has a significant advantage over the YOLOv8n-pose model in predicting the accuracy of tomato picking points.

In summary, the YOLOv8-TP model performs well in synchronous recognition of tomato skewers and their picking points, effectively compensating for the shortcomings of traditional methods.

### 3.2 Comparison of experimental results of mainstream models

Without applying data augmentation operations, comparative analysis of different YOLO keypoint detection models shows that the improved YOLOv8-TP algorithm demonstrates superior performance, characterized by fast recognition speed and high accuracy. The results comparison among YOLOv5s6-pose, YOLOv7-thiny-pose, YOLOv7-w6-pose, YOLOv8n-pose, and YOLOv8-TP is presented in **Table 3**.

**Table 3:** Comparison of training data.

Recognition model	P	R	mAP@.5	mAP@.5:.95	F1-score	Flops(G)	FPS	Params (M)
YOLOv5s6-pose	0.487	0.797	0.918	0.808	0.49	10.2	129.7	15.1
YOLOv7-thiny-pose	0.775	0.875	0.875	0.656	0.82	13.2	134.9	6
YOLOv7-w6-pose	0.908	0.864	0.936	0.853	0.88	51.1	35.1	36.6
YOLOv8n-pose	0.892	0.878	0.928	0.864	0.88	8.3	148.6	3.2
YOLOv8-TP	0.898	0.875	0.938	0.884	0.89	7.6	154.7	46.9

**Figure 9:**
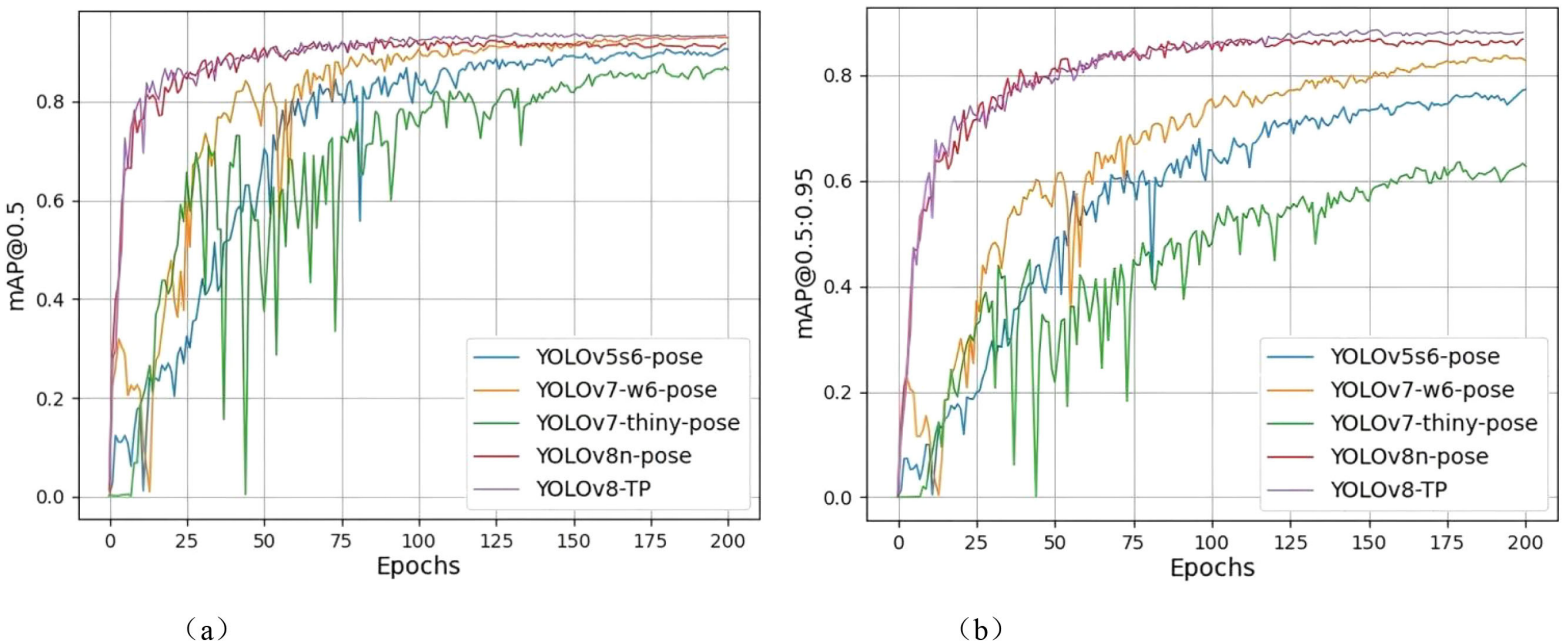
Comparison of mAP@0.5 and mAP@.5:0.95 results. **(a)** mAp@0.5 comparison chart. **(b)** mAp@.5:0.95 comparison chart.

In terms of performance metrics, YOLOv7-w6-pose achieves the highest accuracy of 0.908, followed by YOLOv8-TP with an accuracy of 0.898. The recall values of YOLOv8n-pose and YOLOv8-TP are relatively close, being 0.878 and 0.875 respectively. In terms of the mAP@0.5 metric, the mAP@0.5 of YOLOv8-TP is 0.938, which is 1% higher than that of YOLOv8n-pose. In terms of mAP@0.5:0.95, YOLOv8-TP has the highest value of 0.884, representing a 2% improvement compared to YOLOv8n-pose. Regarding the F1-score metric, YOLOv8-TP reaches a maximum of 0.89, which is a 1% improvement compared to YOLOv8n-pose. In terms of computational resource requirements, YOLOv8-TP has 7.6G Flops, which is 8.1% less than that of the original model YOLOv8n-pose. Moreover, the IoU (Intersection over Union) score of YOLOv8-TP is 0.89, a 2% improvement over the baseline model. This indicates that the predicted bounding boxes of YOLOv8-TP overlap more closely with the ground-truth bounding boxes. As a result, YOLOv8-TP is more precise in localizing small objects and objects in occluded scenarios. As shown in **Figure 9**.

Using detection model evaluation metrics to compare training results, the mAP@0.5 and mAP@0.5:0.95 graphs reveal that the YOLOv7-thiny-pose curve experiences a significant decline between 25 and 75 epochs, exhibiting a highly unstable state, and only gradually reaches a relatively stable state after 100 epochs. The YOLOv5s6-pose curve shows an overall upward trend, with a slight decrease between 75 and 100 epochs before gradually stabilizing. Although the YOLOv7-w6-pose curve performs well in the final results, it demonstrates instability between 0 and 75 epochs, with two significant drops occurring between 0–25 epochs and 50–75 epochs; it then converges to a relatively stable state after 75 epochs. The YOLOv8n-pose curve exhibits a substantial increase before the 75th epoch and gradually reaches a steady state thereafter. In contrast, the YOLOv8-TP curve also shows a rapid increase from 0 to 75 epochs but presents a slow upward trend between 75 and 125 epochs, reaching a relatively stable state at the 125th epoch, with performance achieving an ideal level.

In the mAP@0.5 graph, it is evident that all models have mAP values ranging from 0.8 to 1, with the YOLOv8-TP curve slightly higher than the other four curves. In the mAP@0.5:0.95 graph, only the curves for YOLOv7-w6-pose, YOLOv8n-pose, and YOLOv8-TP exceed 0.8, with YOLOv8-TP yielding better results than the other two models.

In addition to mean average precision (mAP), inference speed (FPS) is also a critical metric for evaluating detection models. In this study, the inference speed of the YOLOv8-TP model reached 154.7 FPS (YOLOv8n-pose The inference speed is 148.6 FPS), which is sufficient to meet the real-time detection requirements of string tomatoes harvesting robots. Therefore, the YOLOv8-TP model demonstrates higher accuracy and faster speed in identifying string tomato fruits and picking points.

### 3.3 Ablation experiment

In this paper, three improvements are made to the original YOLOv8n-pose model (U: Based on the principle of linear stage block, all nonlinear BN layers in the Bottleneck module are removed, a linear scaling layer is introduced instead, and BN layers are added after branching to maintain the diversity of optimization directions. Based on the principle of compressed stage block structure, the linear scaling layer is compressed into the OREPA module to reduce the training cost and maintain high performance, the optimized Bottleneck module is named as Bottleneck-OREPA module, and the C2f module is named as C2f-OREPA module after introducing the C2f module; V: an efficient Partial Self-Attention (PSA) mechanism; W: CGA Fusion is introduced in the Neck network). Each enhancement module is integrated into the original model separately. Ablation experiments are conducted to demonstrate the effectiveness of the enhancement modules, and the corresponding results are shown in **Table 4**.

**Table 4:** Results of ablation experiments.

U^3^	V^4^	W^5^	P	R	mAP@0.5	mAP@0.5:0.95	F1-score	Flops (G)
×	×	×	0.892	0.878	0.928	0.864	0.88	8.3
✓	×	×	0.876	0.892	0.929	0.873	0.87	7.6
×	✓	×	0.905	0.859	0.936	0.885	0.87	8.6
×	×	✓	0.910	0.864	0.935	0.871	0.88	8.6
✓	✓	✓	0.898	0.875	0.938	0.884	0.89	7.6

^4^U: In the Bottleneck module, the nonlinear Batch Normalization (BN) layers are replaced with a linear scaling layer, and a BN layer is added after branching; the linear scaling layer is compressed into the OREPA module. The optimized modules are named as Bottleneck-OREPA module and C2f-OREPA module respectively.

4 V: an efficient Partial Self-Attention ( PSA) mechanism.

5 W: CGA Fusion is introduced in the Neck network.

By replacing C2f with C2f-OREPA, although the Precision slightly decreased compared to YOLOv8n-pose, the Recall, mAP@0.5, mAP@0.5:0.95, and GF lops improved by 1.4%, 0.1%, 0.9%, and 8.1%, respectively. When the PSA self-attention mechanism was individually integrated into the base model, the precision increased by 1.3%, mAP@0.5 by 0.8%, and mAP@0.5:0.95 by 2.1%. This mechanism effectively strengthened the network’s feature extraction capability while suppressing interference from irrelevant information. After individually incorporating CGA Fusion into the Neck network, the precision, mAP@0.5, and mAP@0.5:0.95 increased by 1.8%, 0.7%, and 0.7%, respectively. When PSA and CGA Fusion were introduced simultaneously, mAP@0.5 increased by 1%, clearly demonstrating a synergistic effect between the two: PSA optimizes the overall network performance by enhancing feature extraction and suppressing irrelevant information, while CGA Fusion further improves the model’s robustness to occlusion interference by adaptively enhancing key features. The combination of C2f-OREPA, PSA, and CGA Fusion demonstrated the best overall performance, reducing GF lops by 8.1% while improving Precision, mAP@0.5, mAP@0.5:0.95, and F1-score by 0.6%, 1%, 2%, and 1%, respectively.

**Figure 10:**
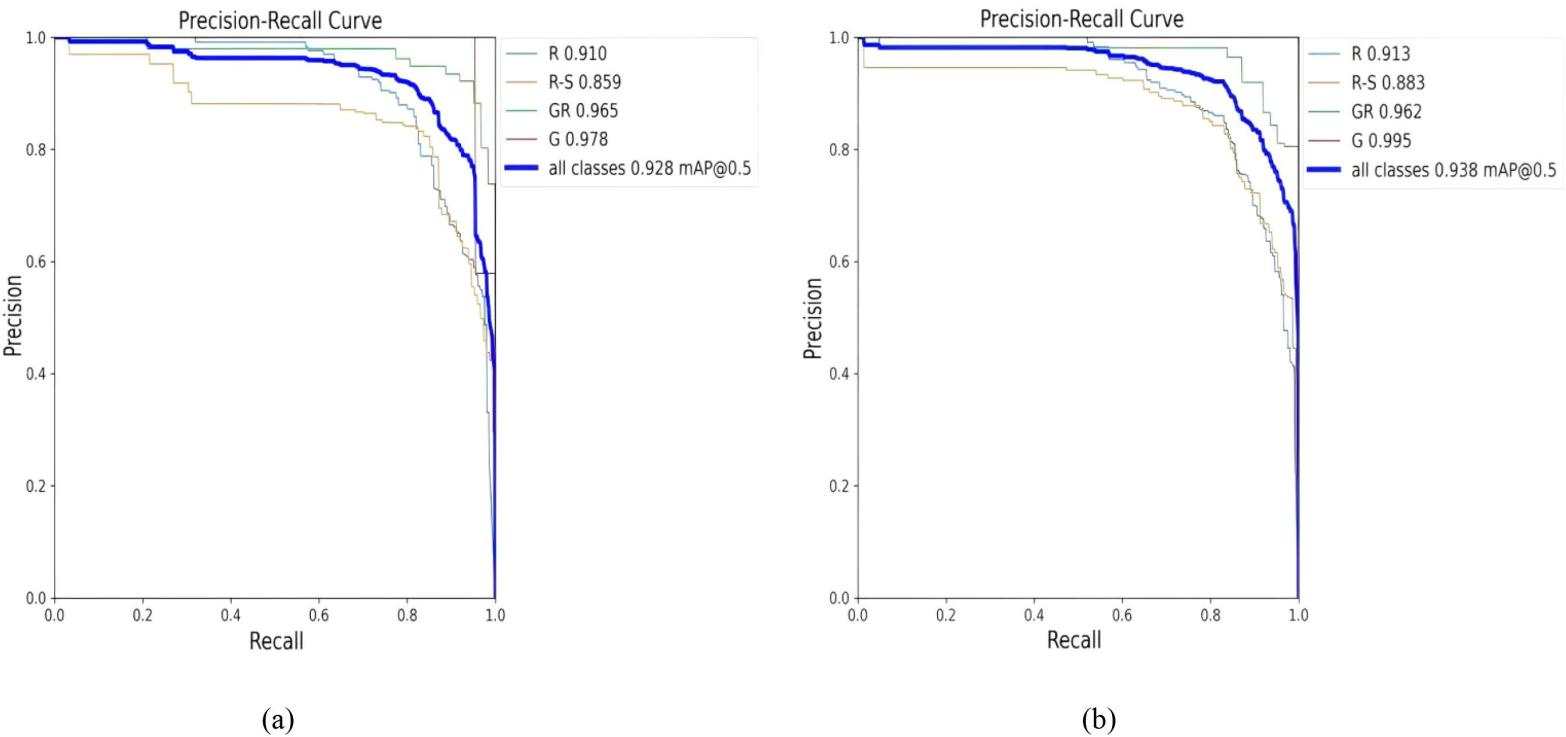
Comparison of YOLOv8n-Pose and YOLOv8-TP RP. **(a)** YOLOv8n-Pose. **(b)** YOLOv8-TP.

The PR curves for the detection and recognition of string tomatoes and picking points using the YOLOv8n-pose model and the YOLOv8-TP model are shown in **Figure 10**. After introducing the OREPA module, PSA self-attention mechanism, and CGA Fusion, the YOLOv8-TP model demonstrated improved performance in detecting and recognizing string tomatoes and their picking points.

### 3.4 Picking point positioning distance error analysis

**Figure 11:**
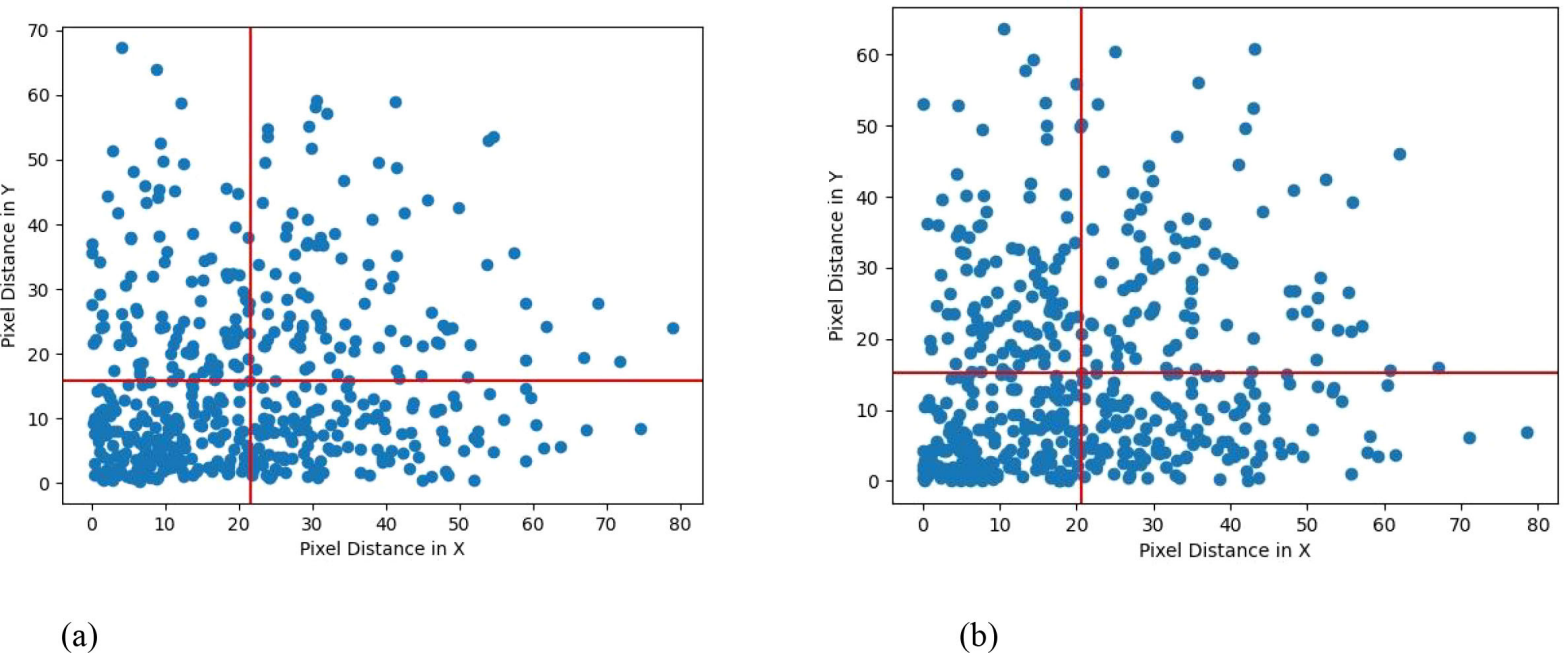
Error scatter distribution of YOLOv8n-Pose and YOLOv8-TP RP. **(a)** YOLOv8n-Pose error scatter distribution. **(b)** YOLOv8-TP error scatter distribution.

**Figure 12:**
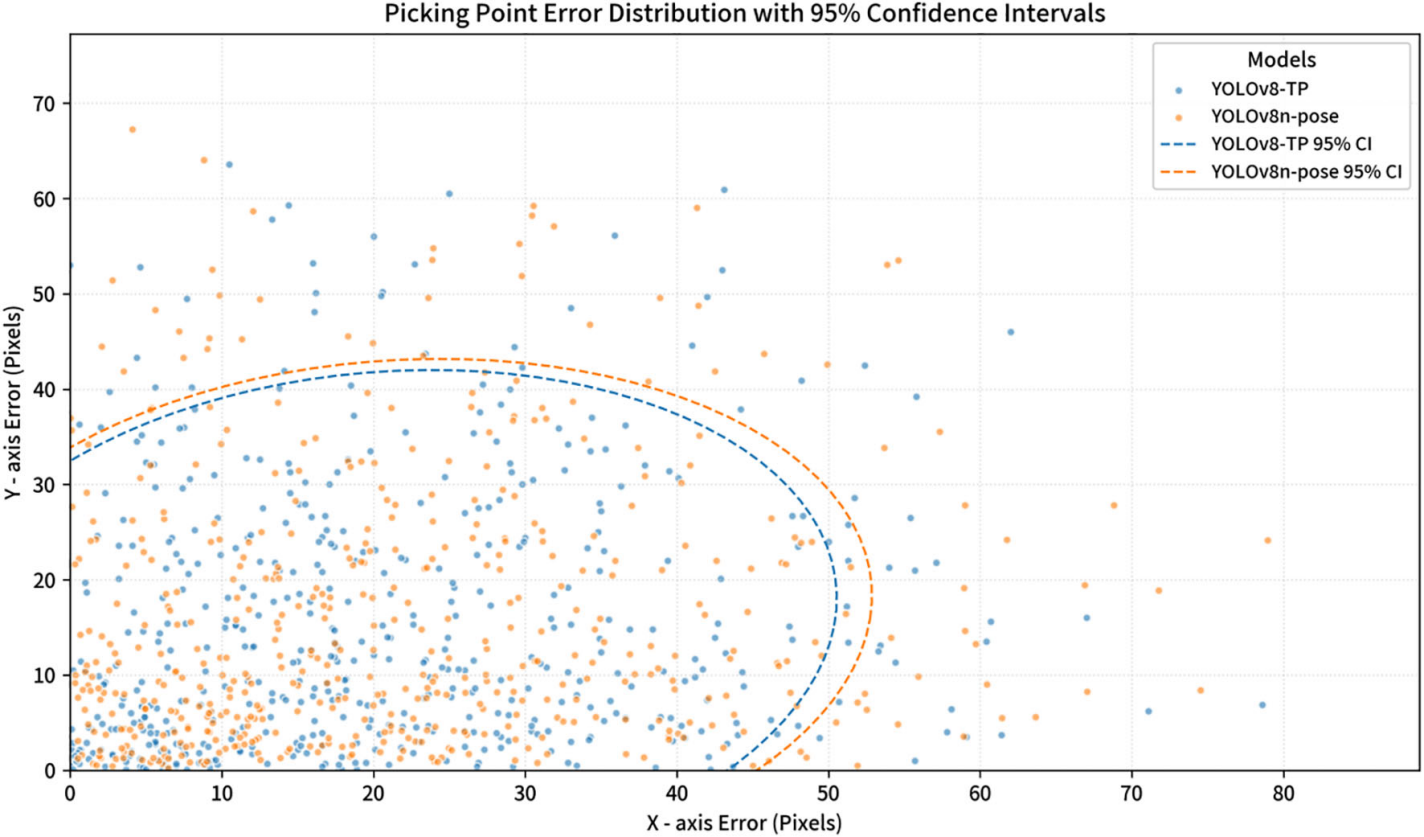
Picking point error distribution of YOLOv8-TP and YOLOv8n-Pose with 95% confidence intervals.


To compare the performance of YOLOv8-TP and YOLOv8n-pose in picking point detection, the average distance error between predicted points and ground truth points was used as an evaluation metric (Guan et al., 2022). The average error was calculated for the coordinates of 500 detected picking points. **Table 5** presents the statistical average distance errors for the 500 predicted picking points from the YOLOv8-TP and YOLOv8n-pose models, along the X-axis, Y-axis, and Euclidean distance. The scatter distribution of errors between the predicted picking points and the ground truth points for YOLOv8-TP is shown in **Figure 11**. Here, the X-axis represents horizontal distance error, while the Y-axis represents vertical distance error. The red vertical line indicates the average horizontal distance error, and the red horizontal line indicates the average vertical distance error. To further visualize the clustering and variance in the error distributions of the YOLOv8-TP and YOLOv8n-pose models, a scatter plot with 95% confidence intervals is presented in **Figure 12**. In this figure, the blue dots represent the error data points of the YOLOv8-TP model, and the orange dots represent those of the YOLOv8n-pose model. The blue and orange curves delineate the 95% confidence intervals for YOLOv8-TP and YOLOv8n-pose, respectively.

**Table 5:** Comparison of average distance errors.

Model	Average distance (pixel)				Standard deviation (pixel)
	X	Y	Euclidean distance	Percentage error (%)	
YOLOv8-TP	20.659	15.177	28.253	5.2	16.686
YOLOv8n-pose	21.577	15.878	29.521	5.8	17.162

(8) $$\left[EP=\frac{❘\text{Predicted}-\text{Actual}❘}{\text{Actual}}\times 100\right]$$

In this paper, Error Percentage (EP) is used to measure the relative error between the model prediction results and the actual sampled values. Its calculation is shown in Equation (8). Error Percentage can intuitively reflect the accuracy performance of the model in practical applications, and the smaller value represents the prediction is closer to the real situation. Regarding the “actual depth”, which is an important benchmark data for model evaluation, this paper obtains it by manual measurement. Specifically, the actual depth of growth of selected fruits was measured at multiple points by the experimenter on site using a scale and other measuring tools while the tomato images were collected, and the average value was taken as the ground truth data. This method ensures the accuracy and reliability of the comparative assessment.

From **Table 5**, it can be observed that the YOLOv8-TP model achieves an Euclidean pixel distance of 28.253 pixels when locating picking points, surpassing the YOLOv8n-pose model’s Euclidean distance of 29.521 pixels. **Figure 11** illustrates that most of the predicted points by YOLOv8-TP are located below and to the left of the average distance error line, indicating that the distance errors in both the X-axis and Y-axis directions are less than 25 pixels. The standard deviation of the Euclidean distance is 16.686 pixels, suggesting that the model exhibits minimal performance fluctuation in picking point localization. Additionally, in practical harvesting applications, based on parameters such as camera resolution, we employed professional conversion methods to conduct rigorous calculation and analysis and determined that the actual physical distance of the position deviation is 0.0265 meters. Considering that the end-effector of the robot has a certain degree of fault tolerance, during actual operations, it can tolerate a position deviation of up to 0.03 meters, thus meeting the harvesting requirements. To sum up, the prediction accuracy of YOLOv8 - TP is sufficient to meet the demands of actual string tomato harvesting.

### 3.5 Get in-depth information on picking sites

In the application of tomato harvesting robots (Ruzhun et al., 2022), accurately obtaining the depth information of picking points is crucial. This information provides the robot with positional data in three-dimensional space, enabling more precise harvesting operations (Arad et al., 2020; Longsheng et al., 2020). The depth camera used in this study is the Intel RealSense D435i, a consumer-grade depth camera developed by Intel, which consists of an RGB camera, two infrared cameras, and an infrared emitter, along with an IMU unit. The ranging principle is similar to that of stereo cameras, relying on the disparity between left and right images to determine distance. The infrared cameras capture the returning infrared light from the target to generate depth images.

**Figure 13:**
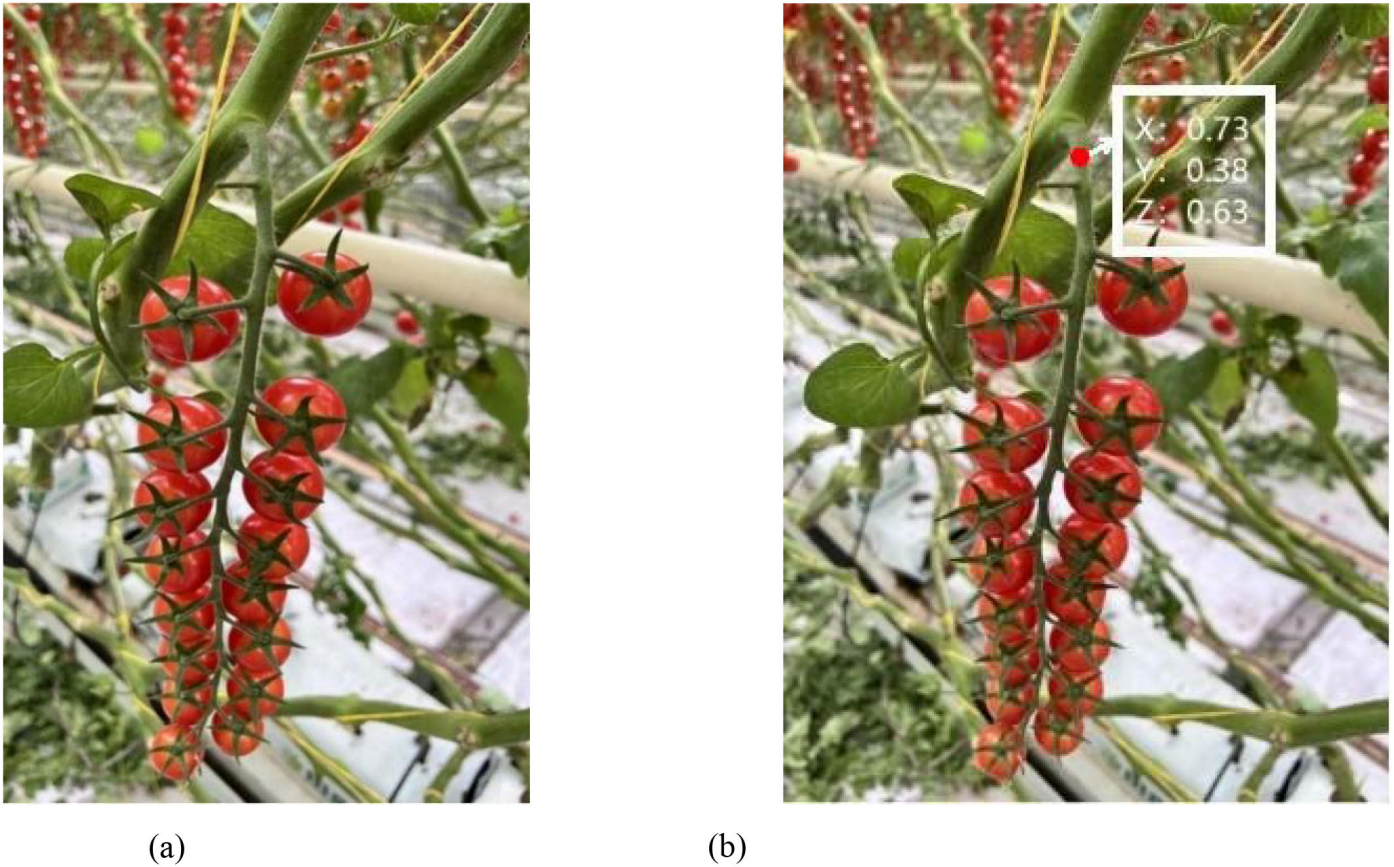
Picking points to obtain depth values. **(a)** original figure. **(b)** Depth value and coordinate information display map.

To obtain the three-dimensional coordinates of the picking points, we converted the two-dimensional coordinates of the detected picking points from previous experiments into coordinates within the camera coordinate system. The D435i depth camera was used to capture images at these locations to acquire depth information. In the depth images, we identified the corresponding pixel locations and read the depth values at those positions, as illustrated in **Figure 13**.

**Figure 14:**
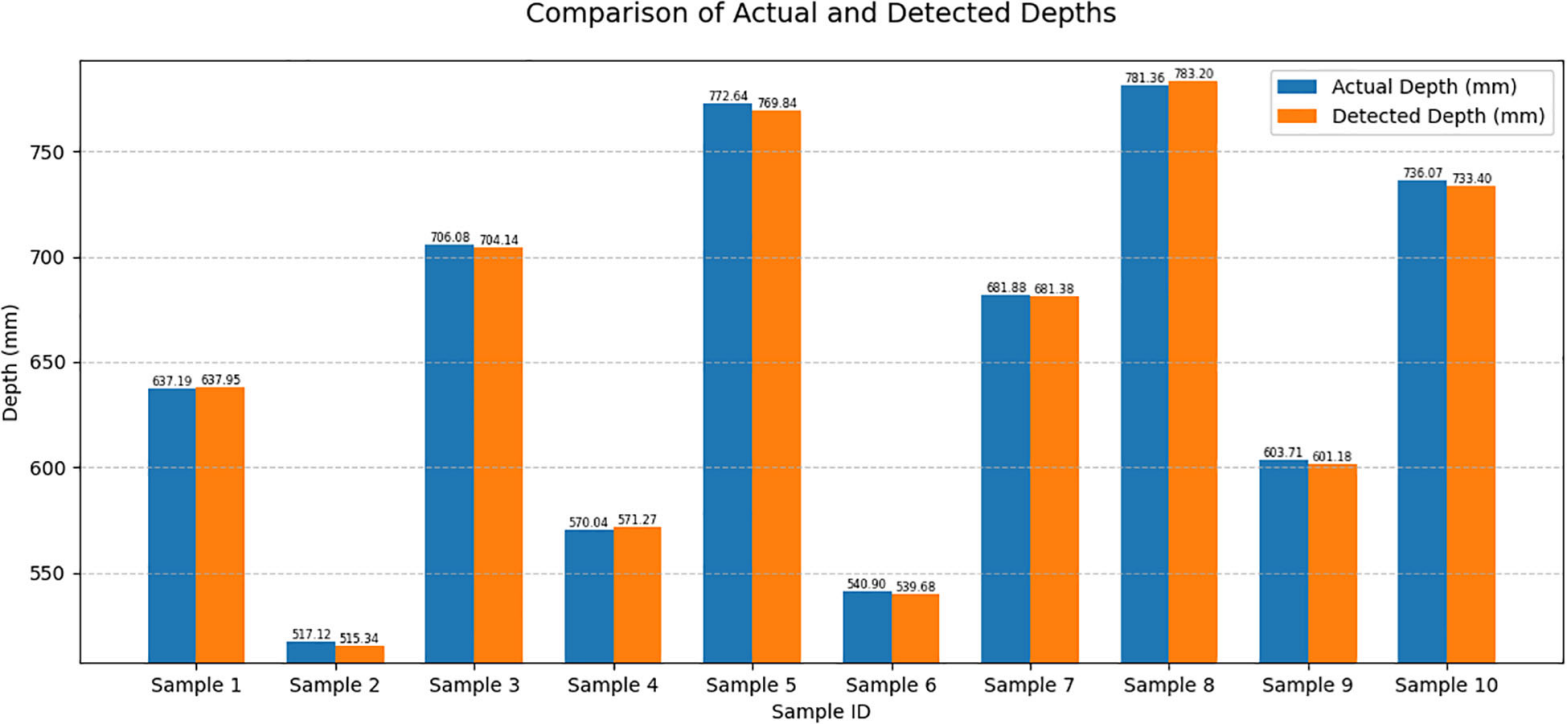
Comparison of actual and detected depths.

The distance between the picking point and the shear center point of the end-effector during the shearing process was used as an evaluation criterion to test the positioning accuracy of the string tomato picking robot (Gong et al., 2022) at the picking point. The depth values of the picking points extracted from the images were validated through comparative analysis with ground-truth measurements. The depth error was quantified as the algebraic difference between the detected and actual depth values, whereas the relative depth error was calculated as the absolute discrepancy normalized by the actual depth, expressed as a percentage. Results showed that the depth error ranged within ±3 mm, corresponding to a relative depth error of 0.073%–0.419%. The comparison between the detected and actual depths is depicted in **Figure 14**, and the corresponding error analysis is presented in **Table 6**.

**Table 6:** Depth value error analysis.

z*/mm actual depth value	z/mm captured depth value	ϵ(z) /mm Depth value error	ϵ(z) /% Depth value error
637.19	637.95	-0.76	0.119
517.12	515.34	1.78	0.344
706.08	704.14	1.94	0.275
570.04	571.27	-1.23	0.216
772.64	769.84	2.8	0.362
540.9	539.68	1.22	0.226
681.88	681.38	0.5	0.073
781.36	783.2	-1.84	0.235
603.71	601.18	2.53	0.419
736.07	733.4	2.67	0.363

## 4 Conclusions

In this study, an improved YOLOv8n-Pose model (named YOLOv8-TP) is proposed to address the challenge of detecting tomato bunch picking points in complex agricultural environments. Improvements include the design of the C2f-OREPA module, the introduction of the PSA mechanism, and the integration of CG Fusion. These improvements greatly reduce the computational requirements of the model, improve the detection accuracy, make the detection of keypoints more robust for small targets/obstructed scenes, and enhance the extraction of key features, effectively overcoming the limitations of the traditional approach to localizing the picking points in the case of occlusion and changing environments.

Compared with existing detection methods for string tomato picking points, the YOLOv8-TP model proposed in this study exhibits significant advantages in the following aspects:

1. Architecture design: Traditional instance segmentation methods [e.g., the two-stream algorithm of (Rong et al., 2025)] require stage-wise processing of RGB and depth data, with high computational complexity (Flops=22.4G). These methods are sensitive to occluded scenes, wherein the detection error increases by more than 15%. In contrast, YOLOv8-TP adopts an end-to-end key-point detection architecture. Through the lightweight design of the C2F-ORepa module (Flops=7.6G, representing an 8.1% reduction) and the PSA global attention mechanism, it achieves multi-scale feature fusion and enhanced occlusion robustness.

2. Detection efficiency: The 3D pose detection method of Ci et al (Zhang et al., 2022). relies on multi-stage key-point prediction, with a reasoning speed of only 35 FPS (frames per second), which struggles to meet real-time requirements. YOLOv8-TP, enhanced by the CGA-Fusion adaptive feature mechanism, maintains a detection accuracy of 89.8% while enhancing the reasoning speed to 154.7 FPS, satisfying the real-time operation requirements of robots.

3. Synchronous detection capability: Traditional methods require separate fruit detection and picking-point positioning [e.g., the ROI screening method of (Zhou et al., 2023)], leading to a cumulative error of 5.8%. This model achieves synchronous positioning through joint optimization of detection and key-point regression. The Euclidean distance error is 28.25 pixels (below the 25-pixel threshold), and the depth error is <3 mm, and the positioning accuracy is improved by 12%.

The comparison demonstrates that through its lightweight architecture and multi-mechanism integration, YOLOv8-TP significantly outperforms traditional methods in detection efficiency and accuracy within complex environments, providing a superior solution for the automatic picking of string tomatoes.

Compared with traditional methods, the end-to-end design of the model avoids redundant computations at multiple stages, and the FPS of the model can be stabilized at around 33 on the pickup robot, while the FPS of some traditional methods (Openpose) is only in the single digits, which simplifies the process of deploying the device at the edge of the operation; at the same time, there is the disadvantage of relying on high-quality labeled data, which is more sensitive to human labeling errors. YOLOv8-TP model exhibits excellent performance in detecting tomato bunch picking points and demonstrates the potential for application in other agricultural scenarios. These contributions provide valuable references for the development of advanced agricultural robotic systems. In greenhouse environments, although the proposed YOLOv8 - TP model achieves good results in detecting tomato bunches and their picking points, it still has some limitations. A noteworthy limitation is its performance under extreme lighting conditions in a greenhouse. For example, when there is intense glare or extremely poorly lit areas, the visual information acquired by the camera may be compromised, which may result in less accurate model detection results. Another aspect is that when dealing with highly complex growth postures of string tomatoes, such as cases of extremely tangled or heavily shaded leaves and branches, the model may face challenges in accurately recognizing each tomato and accurately determining the picking point, which may affect picking efficiency. In practical applications of harvesting robots, our approach is at the heart of the visual recognition and localization module. At this stage, we have integrated the model into a prototype tomato harvesting robot. Through initial testing, robots equipped with our model can recognize and localize bunches of tomatoes and their picking points to a certain extent, enabling them to perform basic harvesting operations. However, there are still several areas that need to be improved. For example, the speed and accuracy of model inference need to be further improved to meet the high-efficiency requirements of large-scale harvesting. There is also a need to optimize the coordination between the robot mechanics and the vision system to ensure seamless operation: Adaptability to Different Crops: Future work will focus on extending the model’s applicability to other crops with varying shapes, textures, and environmental conditions. Real-time deployment: Alternative optimization methods can be explored to further reduce inference time for real-time applications in field harvesting scenarios. Integration with multi-sensor systems. Our model demonstrates strong adaptability and can be applied to other crops with different shapes, structures, and environmental conditions. First, the model structure can learn various features in images, including shape, texture, color, and more. Second, we enhance the model’s generalization ability through methods such as data augmentation and transfer learning. For example, we plan to collect images of crops like grapes and cucumbers that include different growth stages, lighting conditions, and angles. Additionally, we can optimize the model for specific crop characteristics, such as enhancing its ability to detect small objects and adjusting the size and proportions of anchor boxes to be more appropriate.Inspired by the work of Deng et al. (Deng et al., 2025). in dealing with accurate geometric localization of free-form surfaces, we plan to design more surface-adaptive and occlusion-resistant localized feature extraction modules to cope with the complex phenological variations of tomato bunches in their natural environments in our future research. In addition we will try to encode 3D spatial geometric priors (e.g., surface continuity, fruit stalk directionality) of tomato and fruit stalks into deep learning models to constrain and improve the accuracy of picking point prediction. By combining the YOLOv8-TP model with other sensors (such as lidar or thermal imaging cameras), drawing on the camera-radar fusion framework, modal interaction, and robust representation ideas in (Liu et al., 2024), we can design a multimodal fusion network suitable for agricultural scenarios. This is expected to further improve recognition and localization accuracy under extreme lighting, severe obstruction, or complex background conditions, thereby meeting the ever-growing demands of smart agriculture and precision agriculture.

In addition, we are optimistic about the open source of the Tomato-P dataset. If you are interested, please contact the corresponding author. This will provide reusable and scalable tools and data resources for the fields of intelligent agriculture and automatic harvesting, promoting technological development and academic exchange in these areas.

## Data Availability

The raw data supporting the conclusions of this article will be made available by the authors, without undue reservation.

## Footnotes

1 BN, Batch Normalization; ReLU, Rectified Linear Unit.

2 MHSA, multi-head self-attention module.

3 U: In the Bottleneck module, the nonlinear Batch Normalization (BN) layers are replaced with a linear scaling layer, and a BN layer is added after branching; the linear scaling layer is compressed into the OREPA module. The optimized modules are named as Bottleneck-OREPA module and C2f-OREPA module respectively.

## References

[B1] Arad B. Balendonck J. Barth R. Ben-Shahar O. Edan Y. Hellström T.. (2020). Development of a sweet pepper harvesting robot. J. Field Robotics 37, 1027–1039. doi: 10.1002/rob.21937

[B2] Bai Y. Mao S. Zhou J. Xu L. Zhang Z. Tang Y.. (2023). Clustered tomato detection and picking point location using machine learning-aided image analysis for automatic robotic harvesting. Precis. Agric. 24, 727–743. doi: 10.1007/s11119-022-09972-6

[B3] Berenstein R. Shahar O. B. Shapiro A. Edan Y. (2010). Grape clusters and foliage detection algorithms for autonomous selective vineyard sprayer. Intelligent Service Robotics 3, 233–243. doi: 10.1007/s11370-010-0078-z

[B4] Chen Z. He Z. Lu Z.-M. (2023). DEA - Net: Single image dehazing based on detail - enhanced convolution and content - guided attention. arXiv preprint arXiv:2301.04805. Available online at: https://arxiv.org/pdf/2301.04805 (Accessed October 25, 2024). 10.1109/TIP.2024.3354108 38252568

[B5] China Report Hall Network. (2024). Analysis of the market prospect of cherry tomatoes in 2024: the output of China’s cherry tomato market will exceed 11 million tons. Available online at: https://m.chinabgao.com/info/1252151.html (Accessed October 30, 2024).

[B6] Deng J. Zhang Z. Lin Q. Wang Y. Liu C. Chen H.. (2025). “A precise method for identifying 3-D circles in freeform surface point clouds,” in IEEE transactions on instrumentation and measurement (Piscataway, NJ, USA: IEEE), 74, 1–13. doi: 10.1109/TIM.2025.3547492

[B7] Fujinaga T. Yasukawa S. Ishii K. (2021). “Evaluation of tomato fruit harvestability for robotic harvesting,” in 2021 IEEE/SICE international symposium on system integration (SII)(Iwaki, Fukushima, Japan: IEEE), 35–39. doi: 10.1109/IEEECONF49454.2021.9382603

[B8] Gong L. Wang W. Wang T. Liu C. (2022). Robotic harvesting of the occluded fruits with a precise shape and position reconstruction approach. J. Field Rob. 39, 69–84. doi: 10.1002/rob.22041

[B9] Guan Z. Li H. Zuo Z. Pan L. (2022). Design a robot system for tomato picking based on YOLO v5. IFAC-PapersOnLine 55, 166–171. doi: 10.1016/j.ifacol.2022.05.029

[B10] Hu M. Feng J. Hua J. Lai B. Huang J. Gong X.. (2022). Online Convolutional Re-parameterization. arXiv preprint arXiv:2204.00826. Available online at: https://arxiv.org/pdf/2204.00826 (Accessed November 13, 2024).

[B11] Klaoudatos D. S. Moulianitis V. C. Aspragathos N. A. (2019). Development of an experimental strawberry harvesting robotic system. ICINCO 2), 437–445. doi: 10.5220/0007934004370445

[B12] Li H. Huang J. Gu Z. He D. Huang J. Wang C. (2024). Positioning of mango picking point using an improved YOLOv8 architecture with object detection and instance segmentation. Biosyst. Eng. 247, 202–220. doi: 10.1016/j.biosystemseng.2024.09.015

[B13] Liu X. Li Z. Zhou Y. Peng Y. Luo J. (2024). Camera–radar fusion with modality interaction and radar gaussian expansion for 3D object detection. Cyborg Bionic Syst. 5, 79. doi: 10.34133/cbsystems.0079, PMID: 40353135 PMC12063725

[B14] Longsheng F. Fangfang G. Jingzhu W. Rui L. Manoj K. Qin Z. (2020). Application of consumer RGB-D cameras for fruit detection and localization in field: A critical review. Comput. Electron. Agric. 177, 105687. doi: 10.1016/j.compag.2020.105687

[B15] Matsuo T. Takemura Y. Sonoda T. Nishida Y. Yasukawa S. Ishii K. (2021). Tomato-harvesting robot competition: aims and developed robot of 6th competitions. Proc. Int. Conf. Artif. Life Robotics 26, 397–400. doi: 10.5954/ICAROB.2021.OS22-2

[B16] Qin Z. Jianmin C. Bin L Can Xu (2021). Method for recognizing and locating tomato cluster picking points based on RGB-D information fusion and target detection. Trans. Chin. Soc. Agric. Eng. (Transactions CSAE) 37, 143–152. doi: 10.11975/j.issn.1002-6819.2021.18.017

[B17] Rong Q. Hu C. Hu X. Xu M. (2023). Picking point recognition for ripe tomatoes using semantic segmentation and morphological processing. Comput. Electron. Agric. 210, 107923. doi: 10.1016/j.compag.2023.107923

[B18] Rong J. Zheng W. Qi Z. Yuan T. Wang P. (2025). RTMFusion: An enhanced dual-stream architecture algorithm fusing RGB and depth features for instance segmentation of tomato organs. Measurement 239, 115484. doi: 10.1016/j.measurement.2024.115484

[B19] Rongli G. Liu Y. Xu G. (2024). TL-YOLOv8: A blueberry fruit detection algorithm based on improved YOLOv8 and transfer learning Vol. 12 (Piscataway, NJ, USA: IEEE Access), 86378–86390. doi: 10.1109/ACCESS.2024.3408881

[B20] Ruzhun Z. Yuchang Z. Yuanhong L. (2022). An end-to-end lightweight model for grape and picking point simultaneous detection. Biosyst. Eng. 223, 174–188. doi: 10.1016/j.biosystemseng.2022.08.013

[B21] Wang A. Chen H. Liu L. Chen K. Lin Z. Han J.. (2024). YOLOv10: Real-Time End-to-End Object Detection. arXiv preprint arXiv:2405.14458. Available online at: https://arxiv.org/pdf/2405.14458 (Accessed December 7, 2024).

[B22] Wang B. Yang M. Cao P. Shang Z. Hu Y. Li X. (2025). A novel embedded cross framework for high-resolution salient object detection. Appl. Intell. 55, 277. doi: 10.1007/s10489-024-06073-x

[B23] Wang Z. Zhu H. (2023). Tomato picking robot based on deep learning. J. Artif. Intell. Pract. 6, 70–73. doi: 10.23977/jaip.2023.060811

[B24] Xu Z. Liu J. Wang J. Cai L. Jin Y. Zhao S.. (2023). Realtime picking point decision algorithm of trellis grape for high-speed robotic cut-and-catch harvesting. Agronomy 13, 1618. doi: 10.3390/agronomy13061618

[B25] Yan Y. Zhang J. Bi Z. Wang P. (2023). “Identification and location method of cherry tomato picking point based on si-YOLO,” in 2023 IEEE 13th international conference on CYBER technology in automation, control, and intelligent systems (CYBER)(Qinhuangdao, China), 373–378. doi: 10.1109/CYBER59472.2023.10256630

[B26] Zhang G. Cao H. Jin Y. Zhong Y. Zhao A. Zou X.. (2024). YOLOv8n-DDA-SAM: accurate cutting-point estimation for robotic cherry-tomato harvesting. Agriculture 14, 1011. doi: 10.3390/agriculture14071011

[B27] Zhang F. Gao J. Zhou H. Zhang J. Zou K. Yuan T. (2022). Three-dimensional pose detection method based on keypoints detection network for tomato bunch. Comput. Electron. Agric. 195, 106824. doi: 10.1016/j.compag.2022.106824

[B28] Zhang Y. Song C. Zhang D. (2020). Deep learning-based object detection improvement for tomato disease. IEEE Access 8, 56607–56614. doi: 10.1109/ACCESS.2020.2982456

[B29] Zhang T. Wu F. Wang M. Chen Z. Li L. Zou X. (2023). Grape-bunch identification and location of picking points on occluded fruit axis based on YOLOv5-GAP. Horticulturae 9, 498. doi: 10.3390/horticulturae9040498

[B30] Zhou L. Cai J. Ding S. (2023). The identification of ice floes and calculation of sea ice concentration based on a deep learning method. Remote Sens. 15, 2663. doi: 10.3390/rs15102663

[B31] Zhou X. Wu F. Zou X. Zhang S. Lin G. Xiong J.. (2023). Method for locating picking points of grape clusters using multi-object recognition. Trans. Chin. Soc. Agric. Eng. (Transactions CSAE) 39, 166–177. doi: 10.11975/j.issn.1002-6819.202309105

[B32] Zhu Y. Li S. Du W. Du Y. Liu P. Li X. (2023). Identification of table grapes in the natural environment based on an improved Yolov5 and localization of picking points. Precis. Agric. 5 (2), 23–34. doi: 10.1007/s11119-023-09992-w

